# Cationic Pyrrolidine/Pyrroline-Substituted Porphyrins as Efficient Photosensitizers against *E. coli*

**DOI:** 10.3390/molecules26020464

**Published:** 2021-01-17

**Authors:** Bruno M. F. Ladeira, Cristina J. Dias, Ana T. P. C. Gomes, Augusto C. Tomé, Maria G. P. M. S. Neves, Nuno M. M. Moura, Adelaide Almeida, M. Amparo F. Faustino

**Affiliations:** 1 LAQV-REQUIMTE, Department of Chemistry, University of Aveiro, 3810-193 Aveiro, Portugal; brunoladeira@ua.pt (B.M.F.L.); cristina.jesus.dias@ua.pt (C.J.D.); actome@ua.pt (A.C.T.); gneves@ua.pt (M.G.P.M.S.N.); 2 CESAM, Department of Chemistry, University of Aveiro, 3810-193 Aveiro, Portugal; ana.peixoto@ua.pt

**Keywords:** porphyrin, cycloaddition, dipolarophile, photodynamic inactivation, photosensitizer, *E. coli*, potassium iodide

## Abstract

New porphyrin–pyrrolidine/pyrroline conjugates were prepared by revisiting 1,3-dipolar cycloaddition reactions between a porphyrinic azomethine ylide and a series of dipolarophiles. Cationic conjugates obtained by alkylation of the pyrrolidine/pyrroline cycloadducts showed ability to generate singlet oxygen and to produce iodine in presence of KI when irradiated with visible light. Some of the cationic derivatives showed photobactericidal properties towards a Gram-negative bioluminescent *E. coli*. In all cases, these features were significantly improved using KI as coadjutant, allowing, under the tested conditions, the photoinactivation of the bacterium until the detection limit of the method with a drastic reduction of the required photosensitizer concentration and irradiation time. The obtained results showed a high correlation between the ability of the cationic porphyrin derivative to produce singlet oxygen and iodine and its *E. coli* photoinactivation profile.

## 1. Introduction

The particular attention given by the scientific community to porphyrin derivatives is associated with the unique physical, chemical and biological features of these macrocycles, responsible by their success in a wide range of applications [1]. The relevance of these molecules in catalysis [2,3,4,5,6], in the design of chemical sensors [7,8,9,10], solar cells [11,12,13,14], new imaging techniques [15,16] and other biomedical applications [17,18] is corroborated by the high number of interdisciplinary studies published each year involving porphyrin motifs. In the medical field, these derivatives are being considered important partners (the photosensitizer) in photodynamic therapy (PDT), a new approach to treat cancer but also other diseases like atherosclerosis, psoriasis and age-related macular degeneration [19,20,21]. More recently, the same principle behind PDT action is being successfully used to eradicate microorganisms, namely multidrug resistant (MDR) bacteria [22,23].

The success of this therapeutic approach, referred to as antimicrobial photodynamic therapy (aPDT), is related, among other factors, to the efficacy of the photosensitizer (PS) after being activated by visible light in the presence of molecular oxygen (O_2_) to produce reactive oxygen species (ROS), namely the highly oxidative singlet oxygen (^1^O_2_). These cytotoxic species are responsible for the damage of cellular components, resulting in the inactivation of microbial species [24,25,26,27,28], through a non-specific mechanism that does not seem to induce the development of resistance [26,29]. Moreover, the extensive research concerning the actuation of porphyrins as photosensitizer agents, towards different microbial species, such as fungi and bacteria (Gram-positive and Gram-negative) in planktonic and biofilm forms, viruses and parasites, shows also the necessity of adapting the treatment protocol, namely in terms of PS structure to the microorganism type [28]. For instance, the best photoinactivation rates of Gram-negative bacteria occur using positively charged PSs, which allows the interaction of the PS with negatively charged sites in liposaccharides belonging to the outer bacterial membrane [23,30,31,32,33].

The efficacy of porphyrinic PSs can be further improved through the introduction of bioactive heterocyclic moieties at different positions of the macrocycle core. Under this context, efficient photoactive molecules have been developed through a suitable insertion of those motifs at β-pyrrolic positions of porphyrin derivatives [34,35]. Pyrrole and pyrrolidine rings have been previously shown to improve the anti-bacterial activity of some natural compounds [36,37,38,39]. This fact prompted us to prepare β-substituted porphyrins with cationic pyrrolidine/pyrroline units and to evaluate their photodynamic efficacy in aPDT. These chemical motifs were introduced by trapping the 1,3-dipole generated from the free-base 2-formyl-5,10,15,20-tetraphenylporphyrin and *N*-methylglycine with various dipolarophiles using a methodology previously developed by our group, but using the corresponding nickel(II) complex [34,40], followed by the cationization of the resulting conjugates.

The synthetic pathway was allowed to obtain directly neutral porphyrin–pyrrolidine/pyrroline and porphyrin–pyrrole conjugates as free-bases. Further quaternization of the porphyrin conjugates afforded four positively charged PSs, whose antibacterial photodynamic activity was assessed against a recombinant bioluminescent *E. coli* strain (a Gram-negative bacterial model) [41]. The photodynamic efficiency of these cationic PSs was also studied in the presence of potassium iodide, a coadjutant compound which has previously been shown to potentiate the activity of some PSs in aPDT [42,43].

## 2. Results and Discussion

### 2.1. Synthesis of Cationic Porphyrinic Conjugates

**Scheme 1:**
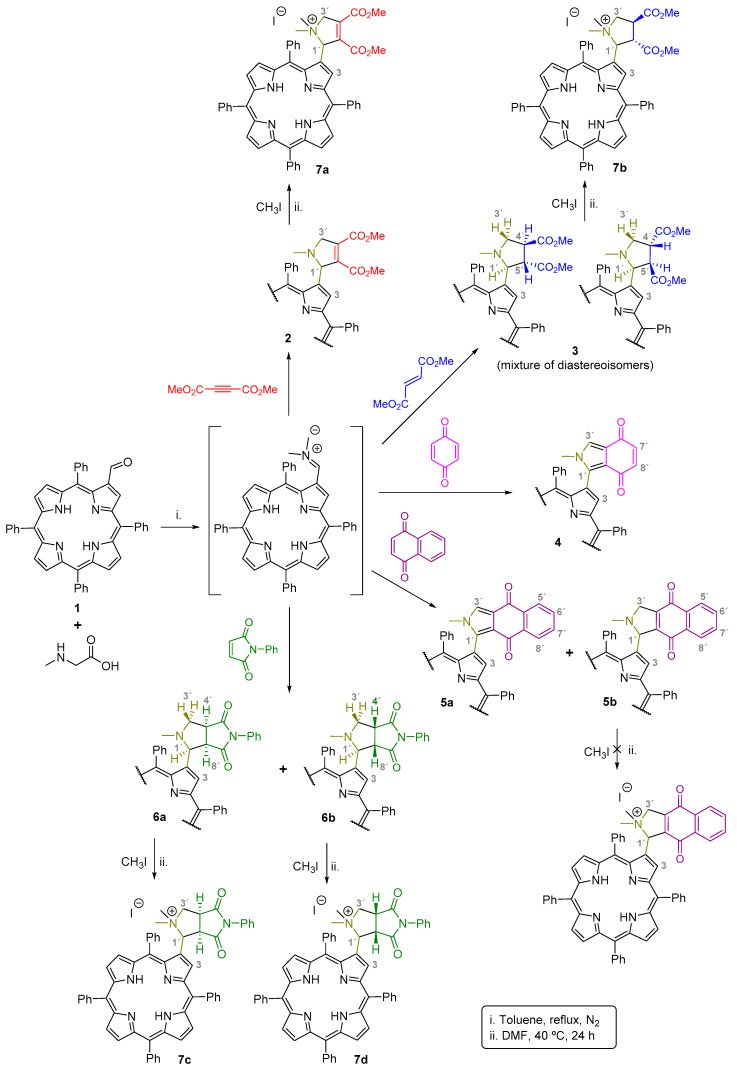
Synthesis of the neutral and cationic porphyrin–heterocycle conjugates **2**–**7**.

As outlined in Scheme 1, the synthesis of the cationic porphyrin–pyrrolidine/pyrroline conjugates required the previous preparation of the corresponding neutral precursors.

The neutral precursors were prepared by trapping the in situ generated azomethine ylide from 2-formyl-5,10,15,20-tetraphenylporphyrin (**1**) and sarcosine with dimethyl acetylenedicarboxylate, dimethyl fumarate, 1,4-benzoquinone, 1,4-naphthoquinone and *N*-phenylmaleimide. All reactions were carried out at in refluxing dry toluene and were stopped when the thin-layer chromatography (TLC) control showed the consumption of the starting porphyrin. After the usual work-up and chromatographic purification, the structures of the conjugates **2**–**6** were confirmed by spectroscopic data (vide infra Section 2.2 and experimental section).

The reactions with dimethyl acetylenedicarboxylate and dimethyl fumarate afforded the expected porphyrin conjugates **2** and **3** in excellent yields, 81% and 89%, respectively. The reaction with 1,4-benzoquinone afforded only the dehydrogenated cycloadduct derivative **4** that was isolated in 80% yield. With 1,4-naphthoquinone, two compounds were isolated, the most abundant being the pyrrole derivative **5a** (54% yield) and the less abundant one the pyrroline derivative **5b** (20% yield). The reaction with *N*-phenylmaleimide also afforded two products that were separated and identified as the pyrrolidine diastereoisomers **6a** and **6b**. These compounds were obtained in 46% and 35% yield, respectively. It is worth noting that compounds **2**–**6**, which have at least one stereogenic center, were obtained as an inseparable enantiomeric mixture.

Comparing the results obtained with the free-base porphyrin **1** with those previously reported using 2-formyl-5,10,15,20-tetraphenylporphyrinatonickel(II) (NiTPP-2-CHO) [40], there are several interesting and important differences. First, in the previous work the free-base porphyrin derivatives were obtained after a demetallation step. Furthermore, in the reaction of the free-base porphyrin with 1,4-benzoquinone, it was possible to obtain the conjugate **4**, while the attempted demetallation of the corresponding Ni(II) complex only afforded decomposition products. Another interesting difference was observed in the reaction with dimethyl acetylenedicarboxylate. Using the free-base porphyrin **1** the pyrroline adduct **2** was obtained as the sole product and in much better yield than the corresponding Ni(II) adduct when Ni(II) 2-formylporphyrin (NiTPP-2-CHO) was used (81% versus 38% yield). Moreover, the demetallation of the nickel complex of the adduct **2** afforded only the free-base of the pyrrole derivative (see structure in ref. [40]). It can also be noted that the conditions used favor the formation of **5a** over **5b**, while those reported with NiTPP-2-CHO favor the formation of **5b** over **5a** probably due to the lower amount of naphthoquinone required.

The quaternization of the neutral porphyrin–heterocycle conjugates **2**, **3**, **5b**, **6a** and **6b** was performed using methyl iodide as alkylating agent, in *N,N*-dimethylformamide (DMF) at 40 °C, for 24 h as depicted in Scheme 1. With exception of 5b, all conjugates afforded the corresponding cationic derivatives **7a** (75%), **7b** (46%), **7c** (74%) and **7d** (62%) in moderate to very good yields. The structures of cationic conjugates **7a-d** were unambiguously established by spectroscopic data, namely NMR, UV−vis spectroscopy and mass spectrometry techniques. All attempts to perform *N*-alkylation of compound **5b** were unsuccessful, even at a higher temperature (100 °C) using dimethyl sulfate as the alkylating agent. In these attempts the initial conjugate was recovered, so its eventual dehydrogenation to the pyrrolo derivative **5a** can be ruled out.

### 2.2. Structural and Photophysical Characterization of the Porphyrin Conjugates

All neutral and cationic porphyrin–heterocycle conjugates were structurally characterized by ^1^H and ^13^C-NMR techniques, mass spectrometry (MS-ESI) and UV-Vis spectroscopy (See Supporting Information, Figures S1–S38). The ^1^H-NMR spectra of the neutral porphyrin–heterocycle conjugates **2**–**6** present characteristic peaks in the aromatic region due to the resonances of the β-pyrrolic protons, centered from ca. δ 8.6 to 9.0 ppm, and due to the resonances of the *meso*-phenyl groups ranging from ca. δ 7.3 to 8.5 ppm. Furthermore, the ^1^H-NMR spectra of compounds **4**, **5a** and **6a**,**b** exhibit additional signals in the aromatic region due to the resonances of the protons present in the pyrrole-fused quinone units (**4** and **5a**) and in the *N*-phenylimide unit (**6a**,**b**). The peaks observed in the aliphatic region of the ^1^H-NMR spectra of compounds **2**–**6** assigned to the resonances of methoxy, methyl, CH and CH_2_ protons confirm the presence of the pyrrolidine- (**3** and **6a**,**b**), pyrroline- (**2** and **5b**) or pyrrole-type (**4** and **5a**) moieties linked to the β-position of the porphyrin macrocycle and are in agreement with the characterizations previous described by Silva et al. [40]

The success of the *N*-alkylation was also confirmed by the ^1^H-NMR spectra of the cationic porphyrins **7**, which display additional resonances due to the second *N*-methyl group present in pyrrolidinium-type moiety. Thus, compound **7a** presents two distinct singlets at δ 2.9 and 3.0 ppm, each corresponding to the resonance of three protons, while compound **7b** presents only one singlet at δ 2.6 ppm, corresponding to six protons. In the ^1^H-NMR spectra of **7c** and **7d** the resonances of the *N*-methyl groups appear also as distinct singlets at δ 2.88, and 2.74 ppm and at δ 3.40, and 2.83 ppm, respectively. The remaining signals in the ^1^H-NMR spectra present a similar profile to that described for the corresponding neutral conjugates.

The characteristic signal due to the resonance of the inner *N*-H protons can also be observed, as expected, at high fields in a range from ca. δ –2.5 to δ –2.8 ppm, in both neutral and cationic derivatives. ESI-MS spectra confirmed the structures proposed for the conjugates, showing peaks with *m/z* corresponding to the expected [M + H]^+^ and [M]^+^ ions, respectively, for the neutral and cationic conjugates.

**Figure 1:**
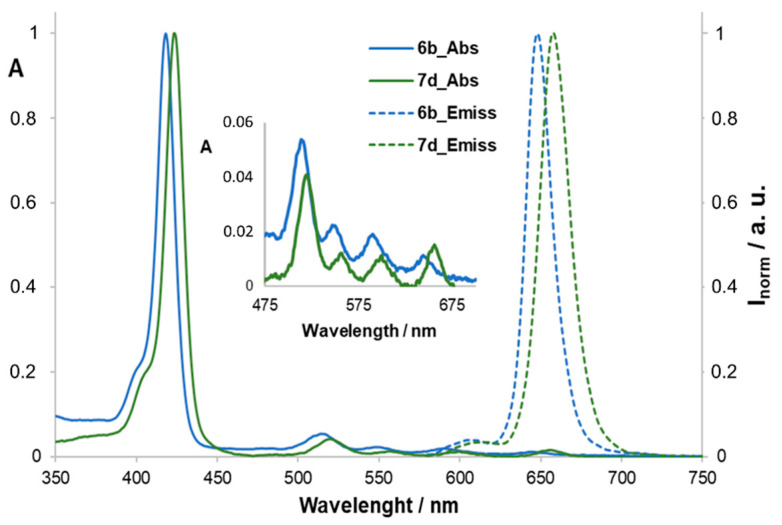
Normalized absorption and emission spectra of conjugates **6b** and **7d** in DMF at 298 K ([**6b**] = [**7d**] = 2.5 µM; λ_exc**6b**_ = 418 nm; λ_exc**7d**_ = 423 nm). The inset shows the absorption at the Q bands region.

Additionally, the absorption and emission spectra of all neutral and cationic conjugates were determined in DMF at 298 K. The absorption and emission spectra of the neutral compound **6b** and of its cationic derivative **7d** are presented, as example, in Figure 1.

The analysis of the absorption spectra reveals a sharp band centered at ca. 420 nm (the Soret band), along with four other less-intense but well-defined bands ranging from 514 to 654 nm, the Q bands [44,45]. These absorption bands are a characteristic feature of free-base *meso*-tetraarylporphyrins. The emission spectra of the compounds present a more intense band in the range between 656–669 nm, along with a much less intense band at ca. 710–715 nm for the neutral compounds and at ca. 620 nm for the cationic compounds.

The absorption and emission spectra also reveal that the introduction of a positive charge in the compounds results in a slight red-shift in the Soret band (*ca.* 3 nm) and in the Q bands (*ca.* 4 nm), as well as a significant red-shift in the bands observed in the emission spectra (5–13 nm).

The fluorescence quantum yields (Φ_F_) of both neutral and cationic compounds were determined through an internal reference method using 5,10,15,20-tetraphenylporphyrin (TPP) as a standard in DMF (Φ_F_ = 0.11) [46,47]. The cationic compounds **7a**–**d** presented lower fluorescence quantum yields when compared to the reference, ranging from 0.06 to 0.08, while the neutral compounds ranged between 0.05 and 0.11, with compound **3** as the only one that matches the quantum yield of TPP (Φ_F_ = 0.11).

### 2.3. Evaluation of Singlet Oxygen Generation

**Figure 2:**
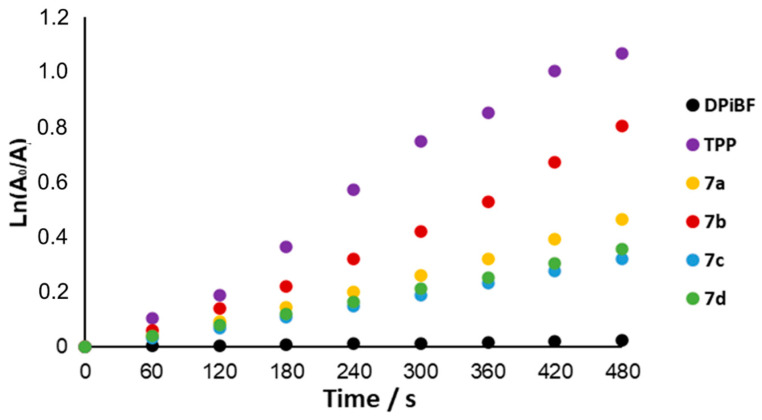
Time-dependent photodecomposition of DPiBF at 50 µM photosensitized by the cationic porphyrin-pyrrolidine conjugates **7a**–**d** at 0.5 µM in DMF upon irradiation with red light (630 ± 20 nm) with an irradiation of 14.0 mW/cm^2^.

To be considered as a photosensitizer candidate in aPDT, the obtained conjugates must be able to generate ROS upon exposure to light, which will induce the inactivation of bacterial cells. Considering that one of the main ROSs responsible for the photodynamic effect is ^1^O_2_ [25], the ability of the conjugates to generate ^1^O_2_ was assessed using an indirect method that monitors the decomposition of 1,3-diphenylisobenzofuran (DPiBF) in the presence of the PS upon irradiation with a red light at 630 ± 20 nm in DMF (Figure 2). The combination of light, porphyrin derivative and oxygen will produce ^1^O_2_, which reacts with DPiBF, thus inducing a decay of the DPiBF absorption band at 415 nm [48,49,50]. 5,10,15,20-Tetraphenylporphyrin (TPP) was used as a reference, once it was known to be a good singlet oxygen generator [51].

The obtained data show that all cationic conjugates can induce the decay of DPiBF absorption when irradiated with light, and no decay is observed in their absence. The best performance to generate ^1^O_2_ was observed for compound **7b**, with an ability of only 20% lower than the reference TPP**.** The remaining adducts exhibit a similar efficiency, although **7a** was slightly better than the other two compounds (**7c**,**d**). These results demonstrated that all derivatives are able to act as PSs in aPDT.

### 2.4. Assessing the Production of Iodine in the Presence of KI

**Figure 3:**
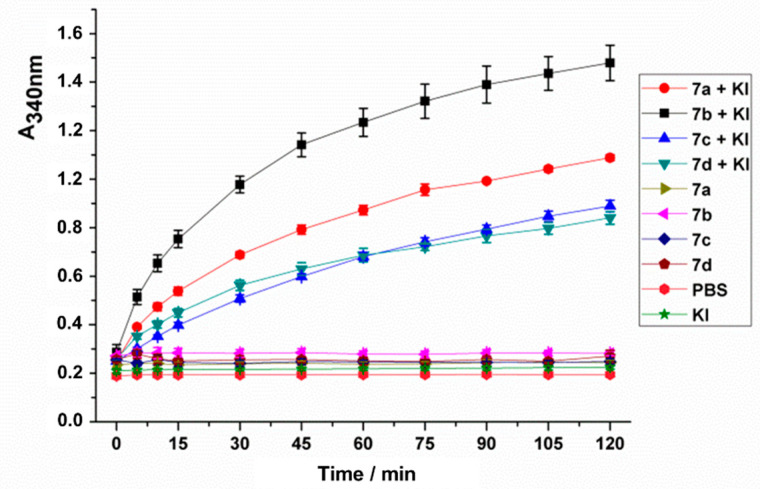
Monitoring of the formation of molecular iodine at 340 nm after different irradiation periods using KI at 100 mM in the presence of each PS (**7a**–**d**) at 5.0 μM and in their absence. Controls where each PS at 5.0 μM was irradiated under the same light protocol but in the absence of KI were also performed.

In order to evaluate if the efficiency of the cationic conjugates in aPDT could be improved by the presence of KI, their ability to produce iodine was assessed. The potentiation effect results from a series of reactions between KI and singlet oxygen, which originates peroxyiodide that can decompose into free iodine or reactive iodine radicals both with oxidative properties [42,52]. Having this in mind, the production of iodine (I_2_) by compounds **7a**–**d** in the presence of KI was assessed by irradiating, with white light (380–700 nm; LED system–25 mW.cm^−2^) for 120 min, a solution of each cationic derivative at 5.0 µM in presence of KI at 100 mM. The absorbance of I_2_ at 340 nm was registered at pre-defined times and the results obtained are summarized in Figure 3 and compared with adequate controls (irradiation under the same light protocol of KI solutions in the absence of the cationic porphyrin conjugates and of the conjugates in the absence of KI). 

The absorbance at 340 nm increased gradually with the irradiation time for all PSs in the presence of KI and was kept stable in the absence of KI, indicating that all conjugates can produce iodine upon irradiation in the presence of KI (Figure 3). The data show that compound **7b** is the most efficient iodine generator, followed by **7a** and then by **7c**,**d**. These results correlate with the efficacy of these conjugates to generate singlet oxygen, **7b** being also the most efficient ^1^O_2_ producer, followed by **7a** and then **7c**,**d**. These results corroborate the I_2_ formation mechanism, which is highly dependent on the ^1^O_2_ formed [42].

### 2.5. Photodynamic Inactivation of E. coli with Cationic Porphyrin–Pyrrolidine Conjugates

The antibacterial photodynamic activity of the cationic porphyrin–pyrrolidine conjugates **7a**–**d** was studied against a bioluminescent *E. coli* bacterial model. The bioluminescence methodology provides a sensitive procedure to monitor, in real-time, the viability of microorganisms, making the aPDT assays rapid, accurate and cost-effective. The strong correlation between CFU mL^−1^ and the bioluminescent signal of the bioluminescent *E. coli* used in this work has already been proved and described [41]. 

**Figure 4:**
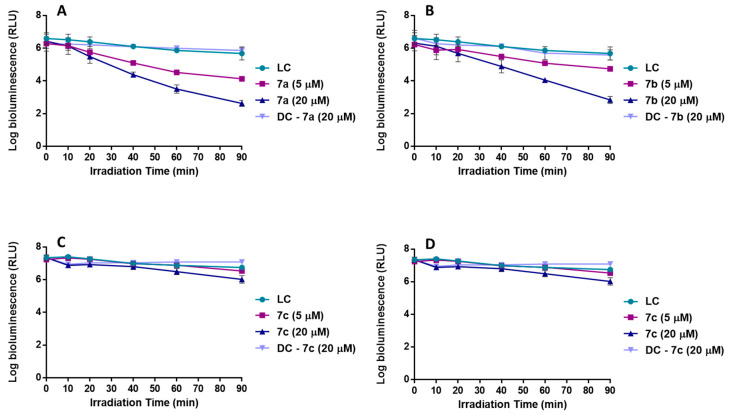
Photodynamic inactivation of bioluminescent *E. coli*, in the presence of compounds **7a** (**A**), **7b** (**B**), **7c** (**C**) and **7d** (**D**) at 5.0 and 20.0 μM, after exposure to white light at 25 mW.cm^−2^ for 90 min. Values are the mean of three independent experiments and the error bars indicate the standard deviation.

**Figure 5:**
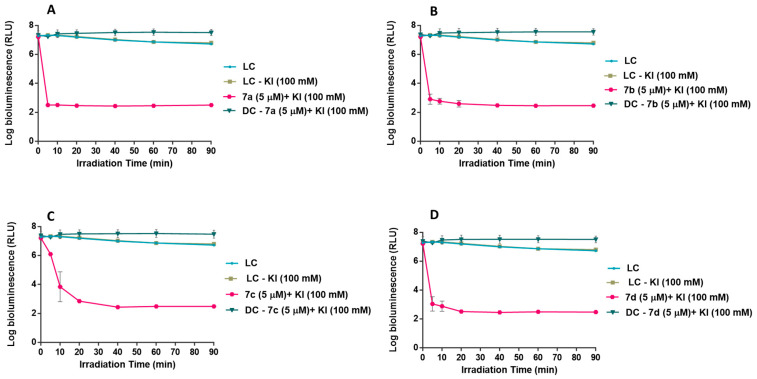
Photodynamic inactivation of bioluminescent *E. coli*, in the presence of compounds **7a** (**A**), **7b** (**B**), **7c** (**C**) and **7d** (**D**) at 5.0 μM in the presence of KI at 100 mM, after exposure to white light at 25 mW.cm^−2^ for 90 min. Values are the mean of three independent experiments and the error bars indicate the standard deviation.

The photoinactivation of *E. coli* in the presence of each cationic derivative **7a**–**d** was evaluated at two different concentrations, 5.0 and 20 μM (Figure 4) and with the PSs at 5.0 μM, the assays were also performed in the presence of 100 mM of KI (Figure 5). This KI concentration was already demonstrated to be non-toxic for the microorganisms [42,43,52,53]. These experiments were performed under white light (380–700 nm) at an irradiance of 25 mW cm^−2^ for 90 min. The results obtained showed that all PSs have no toxicity in the dark at the highest tested concentration (20 µM) and neither affect the viability of *E. coli* when combined with KI without irradiation (Figure 4 and Figure 5). The results also demonstrated that the light conditions used in the aPDT experiments and KI at 100 mM do not affect the viability of the bacterial cells. 

The cationic porphyrin conjugates **7a** and **7b** (Figure 4A,B) proved to be the most efficient PSs in the absence of KI, inducing a decrease of 1.5 log_10_ and 0.9 log_10_ (ANOVA, *p* < 0.05) (> 90% reduction), respectively, in the viability of *E. coli* at the lowest tested concentration (5.0 µM) after 90 min of light exposure. At the highest tested concentration (20 µM), **7a** and **7b** promoted a decrease in the bioluminescent signal of 3.1 and 2.8 log_10_, (> 99.9%) respectively (ANOVA, *p* < 0.05). These results also showed that the aPDT effect of the tested PS is directly proportional to the PS concentration increase. On the other hand, the cationic porphyrin–pyrrolidine conjugates **7c** and **7d** (Figure 4C,D) were shown to be the less efficient PSs in the photoinactivation of *E. coli*, not promoting a significant reduction (ANOVA, *p* > 0.05) on the viability of the Gram-negative bacterium at 5.0 μM after 90 min of aPDT treatment. Even at the highest concentration tested (20 μM), the reduction of the bioluminescent signal did not go beyond of 0.7 and 0.4 log_10_ (ANOVA, *p* < 0.05) for **7c** and **7d** respectively. These results are consistent with the data regarding the efficiency of these compounds to generate ^1^O_2_, where the two least efficient producers of ^1^O_2_, PSs **7c** and **7d**, were also shown to be the less efficient PSs.

However, the results achieved with the cationic porphyrin–pyrrolidine conjugates **7a**–**d** at 5.0 μM combined with KI at 100 mM (Figure 5) showed a drastic reduction in the viability of bioluminescent *E. coli*. For all combinations of PS + KI, the detection limit of the methodology was achieved after 20 min of irradiation, resulting in a decrease of 4.2 log_10_ (ANOVA, *p* < 0.05) in the bioluminescent signal. In fact, all the PSs at 5.0 µM combined with KI at 100 mM produced a more significant decrease in bacterial cell viability than each PS at 20.0 µM in the absence of KI.

These results showed that the aPDT effect of cationic derivatives **7a**–**d** against *E. coli* increases with the concentration of the PSs and their efficiency is highly improved in the presence of KI. Trying to correlate the aPDT effect of each PS with the ^1^O_2_ production, conjugates **7c** and **7d** were less effective in the ^1^O_2_ generation, which can justify the poor ability to affect *E. coli* viability. On the other hand, PSs **7a** and **7b** were the best ^1^O_2_ generators and the most efficient PSs in *E. coli* inactivation. It is also important to highlight that the results were achieved with the combination of derivatives **7a**, **7b** and **7d** with KI (Figure 5A,B), where the inactivation profile suffers a more pronounced decrease in the first 5 min of irradiation. This can be explained by the rapid formation of I_2_ (as seen in Figure 3), due to the high capability of this compound to generate ^1^O_2_. 

Moreover, we can also assume that the fact that the positive charge of each compound is not located at the periphery of the macrocycle, hampering the interaction of the PS with the bacterial membrane, can justify the poor aPDT effect when these compounds act alone as PSs. This effect is more pronounced for derivatives **7c** and **7d**, where the bulkier *N*-phenylimide group can compromise this interaction. This structural disadvantage is overcome when the PSs are combined with KI, due the formation of I_2_ mediated by the capability of these compounds to generate ^1^O_2._ Under these conditions, all the PSs are highly effective in the inactivation of *E. coli*. and the interaction between the PS and the bacterial membrane does not seem to be an impediment to efficient photodynamic inactivation. 

## 3. Materials and Methods

### 3.1. General Remarks

^1^H and ^13^C-NMR spectra were recorded on a Bruker Avance 300 (300.13 MHz and 75.47 MHz) ((Bruker, Wissembourg, France) and 500 (500.13 MHz and 125.76 MHz) spectrometer ((Bruker, Wissembourg, France) s, using CDCl_3_ as a solvent and tetramethylsilane as an internal standard. Chemical shifts (δ) are expressed in parts per million (ppm) and coupling constants (*J*) have been expressed in Hertz (Hz). ESI spectra were recorded on a Micromass Q-Tof 2 spectrometer (Micromass, Manchester, UK) operating in positive mode. High resolution mass spectra were recorded on a LTQ Orbitrap XL mass spectrometer (Thermo Fischer Scientific, Bremen, Germany). UV-Vis spectra were recorded using a Shimadzu UV-2501-PC spectrophotometer (Shimatzu, kioto, Japan) and the fluorescence emission spectra were recorded on a Jasco FP-8300 spectrofluorometer (JASCO, Pfungstadt, Germany), using DMF as solvent. Preparative thin-layer chromatography (TLC) was carried out on 20 × 20 cm glass plates coated with silica gel 60 (Merck, 0.5 mm). Analytical TLC was carried out on precoated sheets with silica gel 60 (Merck, 0.2 mm). Bioluminescence readings were performed in a TD-20/20 luminometer (Turner Designs, Inc., Madison, WI, USA).

### 3.2. Synthesis

#### 3.2.1. Synthesis of the Porphyrin Precursor **1**

2-Formyl-5,10,15,20-tetraphenylporphyrin (**1**) was prepared from 5,10,15,20-tetraphenylporphyrinatocopper(II), phosphorus oxychloride and DMF, according to a well established procedure [54].

#### 3.2.2. Synthesis of Neutral Porphyrin Conjugates **2**, **3**, **4**, **5a**,**b** and **6a**,**b**

To a round bottom flask, 2-formyl-5,10,15,20-tetraphenylporphyrin (25 mg, 0.039 mmol, 1 equiv.) was added and dissolved in dry toluene (2 mL), along with sarcosine (0.0249 g, 0.28 mmol, 7 equiv.) and the appropriate dipolarophile (dimethyl acetylenedicarboxylate (0.60 mmol, 15 equiv.), dimethyl fumarate (0.60 mmol, 15 equiv.), 1,4-benzoquinone (0.60 mmol, 15 equiv.), 1,4-naphthoquinone (0.48 mmol, 12 equiv.) or *N*-phenylmaleimide (0.12 mmol, 3 equiv.)). The resulting reaction mixture was heated at reflux, with stirring and under a N_2_ atmosphere. For the reaction with 1,4-benzoquinone, once 3.5 h had passed, a further 8 equiv. of sarcosine and 7 equiv. of 1,4-benzoquinone were added. The reaction was stopped when TLC control showed the total consumption of the starting porphyrin. The reaction mixture was allowed to cool to room temperature. The crude mixture was then purified by thin-layer chromatography using a dichloromethane:toluene (1:1) mixture as eluent for the reaction with 1,4-naphthoquinone and CH_2_Cl_2_ for the reactions with dimethyl acetylenedicarboxylate, dimethyl fumarate, 1,4-benzoquinone and *N-*phenylmaleimide.

2-[3,4-bis(methoxycarbonyl)-1-methyl-2,5-dihydropyrrol-2-yl]-5,10,15,20-tetraphenylporphyrin, **2**.

Yield: 80%. ^1^H-NMR, 300 MHz, CDCl_3_: δ –2.70 (2H, s, *N*H), 2.20 (3H, s, *N*CH_3_), 3.16 (3H, s, 3′-OCH_3_), 3.44 (1H, dd, *J* = 14.4 and 6.1 Hz, H-3′), 3.78 (3H, s, 4′-OCH_3_), 4.13 (1H, dd, *J* = 14.4 and 4.8 Hz, H-3´), 4.84 (1H, dd, *J* = 6.1 and 4.8 Hz, H-1´), 7.69–7.83 (12H, m, H_meta,para_-Ph), 8.09–8.21 (8H, m, H_ortho_-Ph), 8.65 (1H, d, *J* = 4.8 Hz, H-β), 8.72–8.85 (5H, m, H-β), 9.04 (1H, s, H-3) ppm. ^13^C-NMR, 125 MHz, CDCl_3_: δ 35.1, 39.8, 50.8, 51.1, 51.5, 51.7, 52.2, 53.0, 60.6, 62.8, 69.2, 84.5, 119.8, 120.05, 120.13, 120.3, 120.4, 120.6, 125.1, 126.5, 126.7, 126.8, 127.0, 127.7, 127.75, 127.83, 127.9, 128.5, 132.4, 133.6, 134.56, 134.59, 134.66, 134.74, 135.0, 139.0, 141.6, 141.8, 142.10, 142.14, 142.2, 142.4, 145.1, 155.2, 163.6, 164.5, 164.7, 166.2, 168.2 ppm. UV-Vis (DMF): λ_max_/nm (log ε): 419 (5.34), 516 (4.03), 548 (3.64), 592 (3.49), 649 (3.31). Emission (DMF): λ_em_/nm: 660, 713. Φ_F_ (DMF): 0.10. HRMS-ESI(+): *m/z* calculated to C_53_H_42_N_5_O_4_ [M + H]^+^ 812.3237; found 812.3221.

2-[3,4-bis(methoxycarbonyl)-1-methyl-2,3,4,5-tetrahydropyrrol-2-yl]-5,10,15,20-tetraphenylporphyrin, **3**.

Yield: 89% (mixture of isomers). ^1^H-NMR, 300 MHz, CDCl_3_: δ −2.73 (2H, s, *N*H), 2.15 (3H, s, *N*CH_3_), 2.18–2.25 (1H, m, H-4´), 3.48–3.72 (4H, m, H-1´, H-3´ and H-5´), 3.75 (3H, s, OCH_3_), 3.81 (3H, s, OCH_3_), 7.66–7.87 (m, 12 H, H_meta,para_-Ph), 8.03 (1H, d, *J* = 7.4 Hz, H_ortho_-Ph), 8.11–8.20 (6H, m, H_ortho_-Ph), 8.41 (1H, d, *J* = 7.4 Hz, H_ortho_-Ph), 8.62–8.64 (1H, m, H-β), 8.71–8.79 (3H, m, H-β), 8.84–8.88 (2H, m, H-β), 8.92 (1H, s, H-3) ppm. ^13^C-NMR, 125 MHz, CDCl_3_: δ 39.6, 40.4, 44.0, 44.4, 50.9, 51.5, 52.1, 52.4, 53.1, 57.5, 58.6, 64.4, 65.7, 67.4, 119.4, 119.5, 120.0, 120.1, 120.2, 120.4, 120.5, 125.3, 126.5, 126.7, 126.80, 126.83, 127.8, 127.9, 128.26, 128.29, 128.6, 129.1, 129.8, 132.8, 133.7, 134.0, 134.6, 134.7, 134.9, 135.2, 137.9, 141.9, 142.3, 142.35, 142.39, 142.5, 172.60, 172.63, 173.3, 174.0 ppm. UV-Vis (DMF): λ_max_/nm (log ε): 417 (5.69), 514 (4.24), 549 (3.81), 589 (3.70), 645 (3.47). Emission (DMF): λ_em_/nm: 656, 711. Φ_F_ (DMF): 0.11. HRMS-ESI(+): *m/z* calculated to C_53_H_44_N_5_O_4_ [M + H]^+^ 814.3388; found 814.3399.

2-(4,7-dihydro-2-methyl-4,7-dioxobenzo[c]pyrrol-1-yl]-5,10,15,20-tetraphenylporphyrin, **4**. 

Yield: 81%. ^1^H-NMR, 300 MHz, CDCl_3_: δ −2.62 (2H, s, *N*H), 3.39 (3H, s, *N*CH_3_), 6.50 and 6.61 (2H, AB system, *J* = 10.3 Hz, H-5′ and H-6′), 7.11 (1 H, s, H-3′), 7.28–7.39 (3H, m, H_meta,para_-Ph), 7.73–7.79 (9H, m, H_meta,para_-Ph), 7.90–7.93 (2H, m, H_ortho_-Ph), 8.20–8.30 (6H, m, H_ortho_-Ph), 8.66 (1H, d, *J* = 5.0 Hz, H-β), 8.76–8.80 (4H, m, H-β), 8.88 (2H, s, H-β) ppm. ^13^C-NMR, 75 MHz, CDCl_3_: δ 35.0, 119.4, 120.2, 120.4, 120.5, 120.6, 121.3, 124.5, 125.9, 126.0, 126.8, 127.8, 127.9, 128.2, 129.5, 130.3, 131.7, 133.0, 134.6, 135.1, 135.3, 138.7, 140.3, 140.6, 141.8, 142.0, 142.1, 181.2, 182.3 ppm. UV-Vis (DMF): λ_max_/nm (log ε): 420 (5.23), 516 (4.10), 553 (3.65), 594 (3.55), 648 (3.44). Emission (DMF): λ_em_/nm: 661, 714. Φ_F_ (DMF): 0.05. HRMS-ESI(+): *m/z* calculated to C_53_H_36_N_5_O_2_ [M + H]^+^ 874.2864; found 874.2869.

2-(1,3,4,9-tetrahydro-2-methyl-4,9-dioxonaphtho[2,3-c]pyrrol-1-yl)-5,10,15,20-tetraphenylporphyrin, **5a**.

**5a:** Yield: 64%. ^1^H-NMR, 500 MHz, CDCl_3_: δ −2.60 (2H, s, *N*H), 3.51 (3H, s, *N*CH_3_), 6.92 (1H, t, *J* = 7.4 Hz, H-7´), 7.22 (1H, t, *J* = 7.4 Hz, H-6´), 7.30–7.33 (2H, m, H-3´and H-5´), 7.49–7.85 (9H, m, H*_meta,para_*-Ph), 7.90 (1H, dd, *J* = 12.6 and 7.4 Hz, H-8´), 7.97 (1H, d, *J* = 7.7 Hz, H*_ortho_*-Ph), 8.14–8.34 (7H, m, H*_ortho_*-Ph), 8.63 (1H, d, *J* = 5.0 Hz, H-β), 8.76–8.80 (3H, m, H-β), 8.82 (1H, s, H-3), 8.88 (2H, s, H-β) ppm. ^13^C-NMR, 125 MHz, CDCl_3_: δ 35.2, 120.37, 120.41, 120.6, 120.7, 122.7, 125.0, 125.8, 125.9, 126.4, 126.5, 126.6, 126.7, 126.8, 126.9, 127.80, 127.83, 127.86, 127.92, 131.8, 132.5, 132.9, 134.0, 134.6, 134.7, 135.0, 135.1, 136.1, 138.7, 140.4, 141.8, 142.1, 142.2, 179.3, 180.5 ppm. UV-Vis (DMF): λ_max_/nm (log ε): 421 (5.39), 518 (4.20), 553 (3.74), 595 (3.64), 651 (3.53). Emission (DMF): λ_em_/nm: 663, 715. Φ_F_ (DMF): 0.09. HRMS-ESI(+): *m/z* calculated to C_57_H_38_N_5_O_2_ [M + H]^+^ 824.3020; found 824.3027.

2-(4,9-dihydro-2-methyl-4,9-dioxonaphtho[2,3-c]pyrrol-1-yl)-5,10,15,20-tetraphenylporphyrin, **5b**.

**5b:** Yield: 20%. ^1^H-NMR, 300 MHz, CDCl_3_: δ −2.65 (2H, m, *N*H) 2.37 (3H, s, *N*CH_3_), 3.68 (1H, dd, *J =* 6.3 and 16.4 Hz, H_trans_-3′), 4.39 (1H, dd, *J* = 4.8 and 16.4 Hz, H_cis_-3′), 4.97 (1H, br s, H-1′), 7.52–7.76 (15H, m, H*_meta,para_*-Ph, H-5´, H-6´and H-7´), 7.87–7.99 (1H, m, H-8´), 8.21–8.27 (7H, m, H*_ortho_*-Ph), 8.48 (1H, d, *J* = 7.5 Hz, H*_ortho_*-Ph) 8.68–8.89 (7H, m, H-β) ppm. ^13^C-NMR, 125 MHz, CDCl_3_: δ 35.2, 39.6, 58.1, 67.2, 119.97, 120.05, 120.13, 120.4, 120.6, 122.7, 125.0, 125.8, 126.0, 126.2, 126.5, 126.7, 126.8, 127.6, 127.7, 127.8, 127.86, 127.91, 128.4, 131.8, 132.5, 132.9, 133.3, 133.5, 134.6, 134.7, 134.9, 135.0, 135.1, 136.1, 140.4, 141.8, 141.9, 142.1, 142.2, 142.3, 142.5, 179.3, 180.5, 183.1 ppm. UV-Vis (DMF): λ_max_/nm (log ε): 420 (5.33), 517 (4.07), 551 (3.64), 593 (3.53), 649 (3.35). Emission (DMF): λ_em_/nm: 662, 715. Φ_F_ (DMF): 0.08. HRMS-ESI(+): *m/z* calculated to C_57_H_40_N_5_O_2_ [M + H]^+^ 826.3182; found 826.3130.

2-(2-methyl-4,6-dioxo-5-phenyl-1,3,3a,4,6,6a-hexahydropyrrolo[3,4-c]pyrrol-1-yl)-5,10,15,20-tetraphenylporphyrin, **6a** and **6b**.

**6a**:Yield: 46%. ^1^H-NMR, 500 MHz, CDCl_3_: δ −2.70 (2H, s, *N*H), 2.25 (s, 3H, *N*CH_3_), 2.42 (1H, dd, *J* = 9.8 and 7.0 Hz, H_cis_-3´), 3.22 (1H, t, *J* = 7.0 Hz, H-4´), 3.36 (1H, t, *J* = 8.8 Hz, H-8´), 3.70 (1H, d, *J* = 9.8 Hz, H_trans_-3´), 3.88 (1H, d, *J* = 8.8 Hz, H-1´), 7.01 (1H, d, *J* = 7.5 Hz, H*_ortho_*-*N*Ph), 7.28 (1H, d, *J* = 7.5 Hz, H*_para_*-*N*Ph), 7.34 (2H, t, *J* = 7.6 Hz, H*_meta_*-*N*Ph), 7.61 (1H, t, *J* = 7.2 Hz, H*_meta_*-Ph), 7.78–7.70 (10 H, m, H*_meta,para_*-Ph), 7.83 (1H, t, *J* = 7.6 Hz, H*_para_*-Ph), 8.11 (2H, t, *J* = 7.7 Hz, H*_ortho_*-Ph), 8.17–8.21 (5H, m, H*_ortho_*-Ph), 8.41 (1H, d, *J* = 7.2 Hz, H*_ortho_*-Ph), 8.55 (1H, d, *J* = 4.9 Hz, H-β), 8.70 (1H, dd, *J* = 4.9 Hz, H-β), 8.72 (1H, dd, *J* = 4.9 Hz, H-β), 8.76 (1H, dd, *J* = 4.9 Hz, H-β), 8.81–8.84 (3H, m, H-β) ppm. ^13^C-NMR, 125 MHz, CDCl_3_: δ 40.3, 44.6, 50.5, 58.2, 68.1, 119.0, 119.9, 120.2, 120.3, 126.45, 126.53, 126.57, 126.64, 126.76, 126.80, 127.7, 127.8, 128.2, 128.7, 128.8, 129.9, 132.0, 132.4, 133.7, 134.5, 134.56, 134.65, 134.7, 141.9, 142.3, 142.6, 142.7, 174.8, 178.5 ppm. UV-Vis (DMF): λ_max_/nm (log ε): 419 (5.25), 516 (3.81), 550 (3.44), 591 (3.29), 647 (3.06). Emission (DMF): λ_em_/nm: 656, 712. Φ_F_ (DMF): 0.07. HRMS-ESI(+): *m/z* calculated to C_57_H_43_N_6_O_2_ [M + H]^+^ 843.3442; found 843.3436.

**6b**: Yield: 35%. ^1^H-NMR, 500 MHz, CDCl_3_: δ −2.65 (2H, s, *N*H), 1.94 (s, 3H, *N*CH_3_), 2.67 (1H, dd, *J* = 10.3 and 6.1 Hz, H_cis_-3´), 3.46 (1H, t, *J* = 9.7 Hz, H-4´),3.65–3.69 (1H, m, H-8´), 3.87–3.93 (2H, m, H_trans_-3´ and H-1´), 7.37 (1H, d, *J* = 7.2 Hz, H*_ortho_*-*N*Ph), 7.41–7.46 (2H, m, H*_para_*-*N*Ph and H*_para_*-Ph), 7.55 (2H, t, *J* = 7.6 Hz, H*_meta_*-*N*Ph), 7.81–7.69 (11H, m, H*_meta,para_*-Ph), 8.09 (1H, d, *J* = 7.6 Hz, H*_ortho_*-Ph), 8.14 (1H, d, *J* = 7.8 Hz, H*_ortho_*-Ph), 8.19–8.24 6, m, H*_ortho_*-Ph), 8.64 (1H, d, *J* = 4.9 Hz, H-β), 8.72 (1H, dd, *J* = 4.9 Hz, H-β), 8.77 (1H, dd, *J* = 4.9 Hz, H-β), 8.82 and 8.85 (1H, AB system, *J* = 4.9 Hz, H-β), 8.90 (1H, s, H-3) ppm. ^13^C-NMR, 125 MHz, CDCl_3_: δ 38.3, 44.3, 55.8, 57.1, 64.1, 120.07, 120.14, 120.3, 125.3, 126.4, 126.6, 126.7, 126.8, 127.3, 127.8, 127.9, 128.2, 128.3, 128.6, 129.07, 129.13, 131.7, 132.0, 132.6, 134.6, 134.7, 134.9, 135.6, 137.9, 141.7, 141.8, 142.2, 142.6, 175.0, 177.4 ppm. UV-Vis (DMF): λ_max_/nm (log ε): 421 (5.31), 518 (3.92), 553 (3.44), 595 (3.37), 651 (3.13). Emission (DMF): λ_em_/nm: 656, 712. Φ_F_ (DMF): 0.08. HRMS-ESI(+): *m/z* calculated to C_57_H_43_N_6_O_2_ [M + H]^+^ 843.3442; found 843.3446.

#### 3.2.3. Methylation of Porphyrin Derivatives **2**, **3** and **6a**,**b**

To a solution of each porphyrin derivative **2**, **3** and **6a,b** in DMF (0.5 mL) in a sealed tube, an excess of methyl iodide (65 equiv.) was added. The mixture was kept under stirring overnight at 40 °C. After this period, the reaction was cooled and diethyl ether was added and the resulting precipitate was filtered, washed with diethyl ether and finally dissolved in a CH_3_OH/CH_2_Cl_2_ (1:9) mixture. The solvent mixture was removed under reduced pressure. The products of the reactions were purified by preparative TLC using CH_2_Cl_2_/methanol (99:1) mixture as eluent and compounds **7a**–**d** were obtained pure after crystallization from CH_2_Cl_2_/hexane.

2-[3,4-bis(methoxycarbonyl)-1,1-dimethyl-2,5-dihydropyrrol-2-yl]-5,10,15,20-tetraphenylporphyrin iodide, **7a**.

Yield: 75%. ^1^H-NMR, 300 Hz, CDCl_3_: δ −2.64 (2H, br s, *N*H), 2.88 (3H, s, *N*CH_3_), 2.96 (3H, s, *N*CH_3_), 3.82 (3H, s, OCH_3_), 3.95 (3H, s, OCH_3_), 4.53 (1H, dd, *J =* 16.9 and 1.7 Hz, H-*3´*), 5.25–5.30 (1H, m, H-3′), 5.82 (1H, d, *J =* 1.7 Hz, H-1′), 7.72–8.06 (12H, m, H_meta,para_-Ph), 8.08–8.29 (8H, m, H_ortho_-Ph), 8.55 (1H, d, *J* = 4.9 Hz, H-β), 8.75–8.81 (3H, m, H-β), 8.88 (1H, d, *J =* 5.0 Hz, H-β), 8.93 and 8.97 (2H, AB system, *J =* 5.0 Hz, H-β) ppm. ^13^C-NMR, 125 MHz, CDCl_3_: δ 45.3, 46.4, 49.6, 51.1, 53.3, 53.7, 55.3, 69.3, 80.4, 118.5, 121.1, 121.3, 122.2, 127.0, 127.8, 128.16, 128.24, 128.4, 128.7, 129.9, 130.5, 132.6, 134.1, 134.7, 135.1, 136.0, 136.3, 140.7, 141.1, 141.3, 141.4, 160.6, 160.7 ppm. UV-Vis (DMF): λ_max_/nm (log ε): 423 (5.06), 521 (3.71), 557 (3.29), 595 (3.18), 654 (3.23). Emission (DMF): λ_em_/nm: 666, 721. Φ_F_ (DMF): 0.07. HRMS-ESI(+): *m/z* calculated to C_54_H_44_N_5_O_4_^+^ (M)^+^ 826.3388; found 826.3394.

2-[3,4-bis(methoxycarbonyl)-1,1-dimethyl-2,3,4,5-tetrahydropyrrol-2-yl]-5,10,15,20-tetraphenylporphyrin iodide, **7b**.

**7b:** Yield: 46%. ^1^H-NMR, 300 Hz, CDCl_3_: δ −2.73 (2H, br s, *N*H), 2.61 (6H, s, *N*CH_3_), 2.85–3.14 (2H, m, H-3′and H-4′), 3.74 (3H, s, OCH_3_), 3.91 (3H, s, OCH_3_), 4.16–4.29 (2H, m, H-3′ and H-5′), 4.60–4.67 (1H, m, H-1′) 7.67–7.93 (12H, m, H_meta,para_-Ph), 8.08–8.21 (8H, m, H_ortho_-Ph), 8.74–8.96 (7H, m, H-β) ppm. 13C-NMR, 125 MHz, CDCl_3_: δ 42.7, 43.6, 49.0, 49.5, 49.6, 52.4, 53.1, 53.2, 53.3, 53.7, 54.0, 63.4, 64.4, 74.2, 75.5, 118.4, 118.5, 121.1, 121.3, 121.6, 121.8, 126.9, 127.0, 127.8, 128.2, 128.4, 128.47, 128.54, 128.8, 129.3, 129.5, 129.8, 130.0, 130.5, 133.5, 134.1, 134.6, 135.4, 136.2, 140.9, 141.0, 141.3, 141.4, 141.5, 169.7, 170.7, 170.8 ppm. UV-Vis (DMF): λ_max_/nm (log ε): 423 (5.28), 519 (4.00), 553 (3.57), 595 (3.49), 654 (3.44). Emission (DMF): λ_em_/nm: 661, 720. Φ_F_ (DMF): 0.08. HRMS-ESI(+): *m/z* calculated to C_54_H_46_N_5_O_4_^+^ (M)^+^ 828.3544; found 828.3553.

2-(2,2-dimethyl-4,6-dioxo-5-phenyl-1,3,3a,4,6,6a-hexahydropyrrolo[3,4-c]pyrrol-1-yl)-5,10,15,20-tetraphenylporphyrin iodide, **7c** and **7d**.

**7c**: Yield: 74%. ^1^H-NMR, 300 MHz, CDCl_3_: δ −2.56 (2H, s, *N*H), 2.74 (3H, s, *N*CH_3_), 2.88 (3H, s, *N*CH_3_), 3.53–3.61 (1H, m, H-3′), 4.63–4.72 (1H, m, H-4′), 4.79–4.96 (3H, m, H-3′, H-8′ and H-1′), 7.51–7.56 (3H, m, H_ortho,para_-*N*Ph), 7.60–7.67 (3H, m, H_meta_-*N*Ph), 7.78–7.88 (11H, m, H_meta,para_-Ph), 8,02 (2H, t, *J* = 7.3, H_meta_-Ph), 8.09–8.37 (7H, m, H_ortho_-Ph), 8,58 (1H, d, *J* = 8.1 Hz, H_ortho_-Ph), 8,72–8.76 (2H, m, H-β), 8,79 (1H, d, *J* = 4,9, H-β), 8,84 (1H, d, *J* = 4,9, H-β), 8.90 (2H, d, *J* = 5,0, H-β), 8.94 (2H, d, *J* = 5,0, H-β), 9.23 (1H, s, H-3) ppm. ^13^C-NMR, 125 MHz, CDCl_3_: δ 42.2, 46.4, 47.4, 49.8, 51.3, 65.7, 74.7, 118.7, 120.7, 121.2, 122.6, 125.5, 126.4, 126.97, 127.04, 127.5, 127.8, 128.1, 128.7, 128.8, 128.9, 129.3, 129.4, 130.3, 130.8, 131.2, 134.6, 134.8, 138.0, 140.3, 141.1, 141.2, 141.5, 170.9, 172.3 ppm. UV-Vis (DMF): λ_max_/nm (log ε): 421 (5.34), 518 (4.03), 553 (3.68), 595 (3.68), 651 (3.63). Emission (DMF): λ_em_/nm: 667, 721. Φ_F_ (DMF): 0.06. HRMS-ESI(+): *m/z* calculated to C_58_H_45_N_6_O_2_^+^ (M)^+^ 857.3599; found 857.3600.

**7d**: Yield: 62%. ^1^H-NMR, 500 MHz, CDCl_3_: δ −2.67 (2H, br s, *N*H), 2.83 (3H, m, *N*CH_3_), 3.40 (3H, m, *N*CH_3_), 4.16 (1H, dd, *J* = 13.0 and 7.5 Hz, H-4′), 4.39 (1H, t, *J* = 9.9 Hz, H_cis_-3′), 4.78–4.83 (1H, m, H-8′),4.89–4.94 (1H, m, H_trans_-3′), 5.20 (1H, d, *J* = 9.6 Hz, H-1′), 6.57 (2H, d, *J* = 7.7, H_ortho_-NPh), 7.04 (2H, t, *J* = 7.7, H_meta_-*N*Ph), 7.18 (1H, t, *J* = 7.7 Hz, H_para_-*N*Ph), 7.83–7.68 (10H, m, H_meta,para_-Ph), 7.99 (2H, t, *J* = 7.6 Hz, H_meta_-Ph), 8.06 (2H, t, *J* = 7.6 Hz, H_ortho_-Ph), 8.14–8.25 (4H, m, H_ortho_-Ph), 8.31 (1H, d, *J* = 8.1 Hz, H_ortho_-Ph), 8.47 (1H, d, *J* = 7.1 Hz, H_ortho_-Ph), 8.62 (1H, s, H-3), 8.75 and 8.77 (2H, AB system, *J* = 4.9 Hz, H-β), 8.86–8.92 (4H, m, H-β) ppm. ^13^C-NMR, 125 MHz, CDCl_3_: δ 44.0, 48.8, 49.9, 56.3, 63.7, 65.9, 74.9, 119.1, 121.1, 121.2, 121.47, 125.53, 126.9, 127.0, 127.7, 128.2, 128.5, 128.6, 128.8, 130.3, 130.8, 132.9, 134.1, 134.6, 135.9, 140.9, 141.1, 141.4, 141.5, 170.5, 173.2 ppm. UV-Vis (DMF): λ_max_/nm (log ε): 423 (5.21), 520 (3.90), 556 (3.44), 598 (3.39), 655 (3.52). Emission (DMF): λ_em_/nm: 669, 720. Φ_F_ (DMF): 0.06. HRMS-ESI(+): *m/z* calculated to C_58_H_45_N_6_O_2_^+^ (M)^+^ 857.3599; found 857.3593.

### 3.3. Singlet Oxygen Generation

Stock solutions in DMF of each cationic compound **7a**–**d** at 0.1 mM and of 1,3-diphenylisobenzofuran (DPiBF) at 10 mM were prepared. A 2.5 mL mixture containing a solution of each porphyrin (0.5 µM) and DPiBF (50 µM) in DMF were irradiated with a red LED board (630 ± 20 nm) at an irradiance of 14 mW/cm^2^, in a glass cuvette, at room temperature and under gentle magnetic stirring. Controls were also prepared, consisting of a 50 µM solution of DPiBF, and another solution, containing DPiBF (50 µM) and TPP (0.5 µM). The absorption decay of DPiBF at 415 nm was measured every minute for 9 min (540 s).

### 3.4. Detection of Iodine Formation

In a 96-well microplate, appropriate volumes of each compound at 5.0 μM and also combinations of each tested compound at 5.0 μM and KI at 100 mM in PBS were maintained under stirring in the dark for 15 min and then irradiated with a white light delivered by a LED system with an irradiance of 25 mW.cm^−2^. The generation of iodine (I_2_) was monitored by measuring the iodine absorbance at 340 nm at different pre-defined irradiation times in a Synergy™ HTX Multi-Mode Microplate Reader from BioTek Instruments.

### 3.5. Antimicrobial Photodynamic Assays

#### 3.5.1. Photosensitizer Stock Solutions

Stock solutions of each of the cationic porphyrin conjugates were prepared at 500 μM in dimethyl sulfoxide (DMSO) and stored in the dark. Before each experiment, each PS solution was sonicated for 15 min at ambient temperature and diluted to the final concentrations in PBS.

#### 3.5.2. Light Source

Irradiations were performed using a LED system, which delivers photosynthetically active radiation (PAR) white light (380–700 nm), at an irradiance of 25 mW.cm^−2^ measured with an energy power meter Coherent FieldMaxII-Top combined with a Coherent PowerSens PS19Q energy 230 sensor.

#### 3.5.3. Bacterial Strains and Growth Conditions

The bioluminescent *E. coli* Top10 [51], genetically transformed by luxCDABE genes of the marine bioluminescent bacterium *Allivibrio fischeri* [55], was grown on tryptic soy agar (TSA, Merck, Darmstadt, Germany) supplemented with 34 mg.mL^−1^ of chloramphenicol and with 50 mg.mL^−1^ of ampicillin. Before each aPDT assay, an isolated colony was transferred to 10 mL of tryptic soy broth medium (TSB, Merck) and it was grown under stirring (120 rpm) at 25 °C overnight. After that period, an aliquot was transferred to 10 mL TSB under the same growth conditions until a stationary growth phase was achieved. An optical density at 600 nm (OD_600_) of 1.6 ± 0.1 corresponded to ≈ 10^8^ colony-forming units per millilitre (CFU.mL^−1^). The correlation between the CFU.mL^−1^ and the bioluminescent signal (in RLUs) of the bioluminescence *E. coli* strain was previously stablished [42].

#### 3.5.4. Antimicrobial Photodynamic Therapy (aPDT) Procedure

A bioluminescent *E. coli* culture growth overnight was 10-fold diluted in phosphate-buffered saline (PBS), pH 7.4, to a final concentration of ~10^6^ CFU mL^−1^, which corresponds approximately to 10^6^ relative light units (RLU). The obtained suspension was equally distributed in sterilized beakers. Then, adequate aliquots of each positively charged photosensitizer (**7a**–**d**) were added to attain the concentrations of 5.0 µM and 20 µM (total volume was 10 mL per beaker). The samples were protected from light using aluminum foil and remained in the dark for 10 min to favor the binding of the PS to the *E. coli* cells. Light controls (LC, comprising only the *E. coli* suspension exposed to the same light protocol) and dark controls (DC, containing *E. coli* suspension incubated with each porphyrin at the maximum tested concentration (20 µM) protected from light) were also prepared. Following the incubation period, the samples and LC were exposed to light irradiation (380–700 nm) at an irradiance of 25 mW.cm^2^ under stirring for 90 min. DC was maintained in the dark during the aPDT procedure. Aliquots of 1.0 mL of the samples, LC and DC were collected at time 0, and after 10, 20, 40, 60 and 90 min of aPDT treatment and the bioluminescence signal was assessed in a TD-20/20 luminometer (Turner Designs, Inc., Madison, WI, USA).

#### 3.5.5. Antimicrobial Photodynamic Therapy (aPDT) in the Presence of KI

A bioluminescent *E. coli* culture growth overnight was tenfold diluted in PBS, pH 7.4, to a final concentration of ~10^7^ CFU mL^−1^ (approximately to 10^7^ relative light units (RLU)). The obtained suspension was equally distributed in sterilized beakers and an appropriate aliquot of each PS was added to obtain the final concentration of 5.0 µM and 100 mM of KI (total volume was 10 mL per beaker). The samples were protected from light using aluminum foil and remained in the dark for 10 min. In addition to a LC and DC (containing *E. coli* suspension incubated with each PS at 5.0 µM and 100 mM of KI), light control with the same concentration of KI (LC + KI) was also prepared in order to evaluate the effect of KI alone and light in the bacterial suspension viability. After the incubation period, the samples and the LC were exposed to light at an irradiance of 25 mW.cm^−2^ under stirring for 90 min. DC was maintained in the dark during the aPDT procedure. Aliquots of 1.0 mL of the samples and controls were collected at time 0, and after 5, 10, 20, 40, 60 and 90 min of aPDT and the bioluminescence signal was assessed in the luminometer.

#### 3.5.6. Statistical

Three independent experiments with three replicates per assay for each condition were done. The statistical analysis was performed with GraphPad Prism (GraphPad Software, San Diego, CA,USA). Normal distributions were checked by the Kolmogorov–Smirnov test and the homogeneity of variance was verified with the Brown Forsythe test. ANOVA and Dunnet’s multiple comparison tests were applied to assess the significance of the tested conditions. A value of *p* < 0.05 was considered significant.

## 4. Conclusions

In this paper, an efficient pathway to obtain new cationic porphyrin–pyrrolidine/pyrroline conjugates was developed. The access to these derivatives required the preparation of adequate neutral precursors, which were obtained by trapping the azomethine ylide generated from the free-base 2-formyl-5,10,15,20-tetraphenylporphyrin and sarcosine with dimethyl acetylenedicarboxylate, dimethyl fumarate, and *N*-phenylmaleimide. The dehydrogenated adducts obtained with 1,4-benzoquinone and 1,4-naphthoquinone were not susceptible to be cationized. The four positively charged compounds showed an ability to generate singlet oxygen and, in the presence of the coadjutant KI, molecular iodine.

Accordingly, the two most efficient producers of these species, **7a** and **7b**, also presented higher bactericidal activity against *E. coli* at the concentration of 20 µM. Furthermore, this work paved the role of KI as a coadjutant in aPDT, as the addition of this salt greatly increased the photodynamic effect of all tested derivatives (**7a**–**d**), reducing the viability of *E. coli* bacterial cells until detection limit, even at 5.0 μM of each PS. In fact, the use of KI allowed a drastic reduction of the PSs concentration (at least 4 times) and of the time to achieve the inactivation of *E. coli* until the detection limit of the methodology.

Cationic porphyrin–pyrrolidine conjugates **7a** and **7b** at 20 μM and all PSs at 5.0 μM, when combined with KI, can be considered antimicrobial agents according to the American Society of Microbiology, which defines bactericidal agents as compounds that can cause a decrease in the bacterial concentration of at least 3.0 log_10_ (99.9%) [49].

## Data Availability

The data presented in this study are available in supplementary material.

## Supplementary Materials

The following are available online, Figures S1 to S37: Copies of ^1^H, ^13^C, 2D NMR and MS spectra of compounds **2**–**7**; Figure S38: UV-Vis and emission spectra of compounds **2**–**5**, **6a** and **7a**–**c**.

## Sample Availability

Samples of the compounds **1-7** are available from the authors.
