# Supplementary material for: Cationic Pyrrolidine/Pyrroline-Substituted Porphyrins as Efficient Photosensitizers against E. coli
Source: Molecules. 2021 Jan 17;26(2):464. doi: 10.3390/molecules26020464 (PMC7829939; doi:10.3390/molecules26020464)
Supplement: Supplementary file 1 [file molecules-26-00464-s001.pdf]

Supporting Information

Cationic pyrrolidine/pyrroline-substituted porphyrins as efficient photosensitizers against *E. coli*

Bruno M. L. Ladeira [1], Cristina J. Dias [1], Ana T. P. C. Gomes [2], Augusto C. Tomé [1], Maria G. P. M .S. Neves [1], Nuno M. M. Moura [1,*], A. Almeida [2,*] and M. Amparo F. Faustino [1,*]

[1] LAQV-REQUIMTE, Department of Chemistry, University of Aveiro, 3810-193, Aveiro, Portugal; brunoladeira@ua.pt (B.L.); cristina.jesus.dias@ua.pt (C.J.D.); gneves@ua.pt (M.G.P.M.S.N.)

[2] CESAM, Department of Chemistry, University of Aveiro, 3810-193, Aveiro, Portugal; ana.peixoto@ua.pt (A.T.P.C.G.)

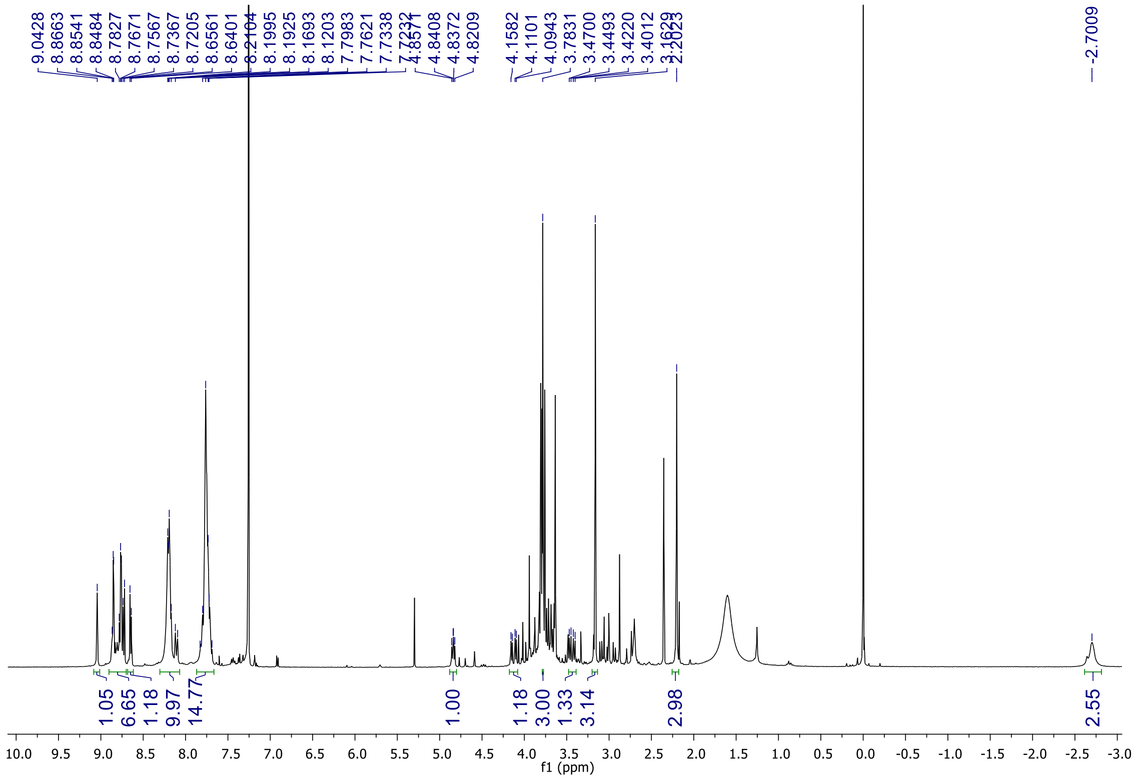

Figure S1. ^{1}H NMR spectrum of compound **2** in $CDCl_3$.

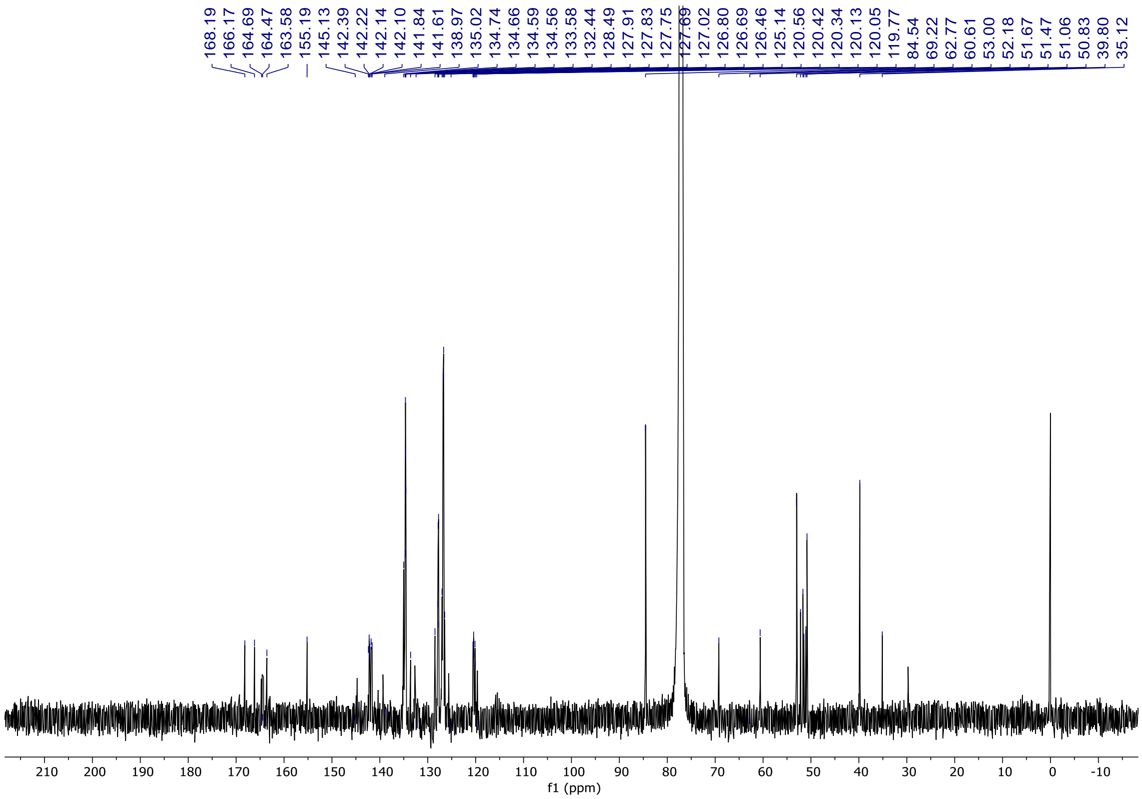

Figure S2. ^{13}C NMR spectrum of compound **2** in $CDCl_3$.

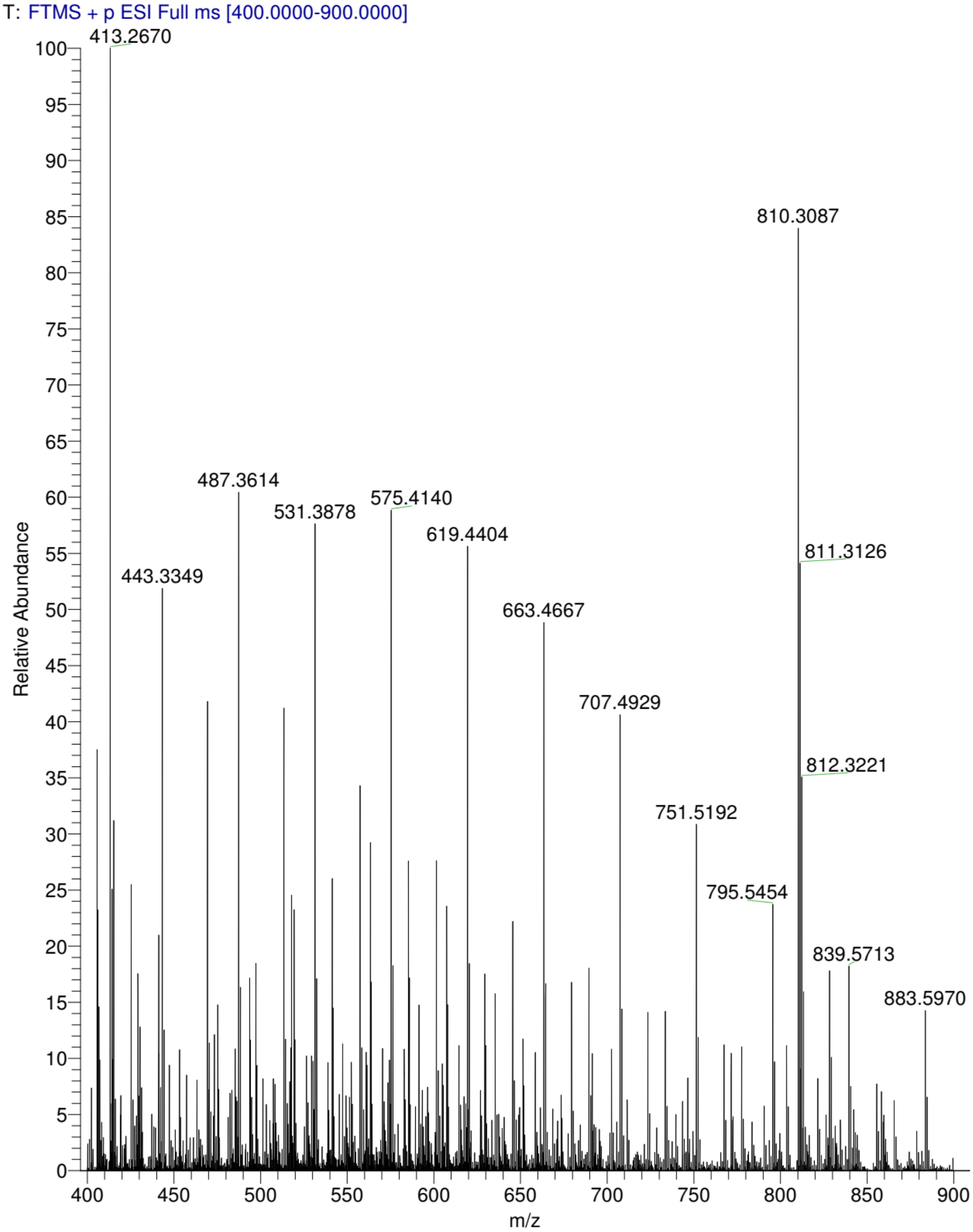

Figure S3. HRMS-ESI(+) spectrum of compound **2**.

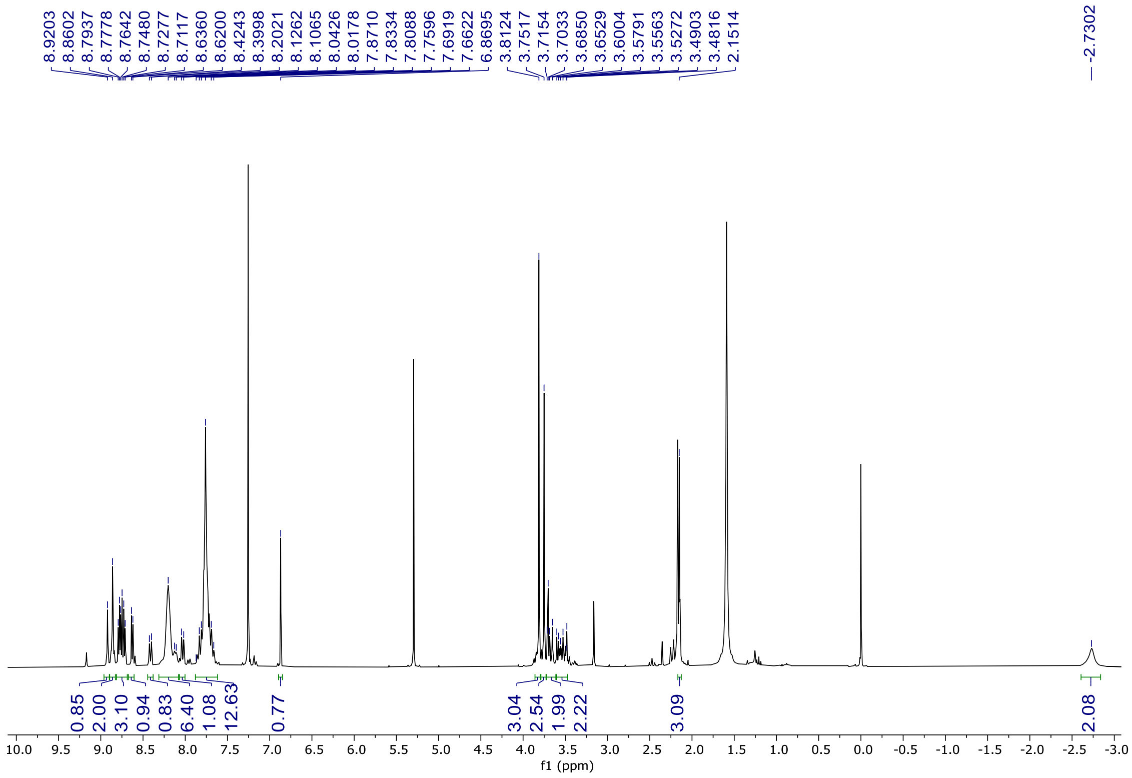

Figure S4. ^{1}H NMR spectrum of compound **3** in $CDCl_3$.

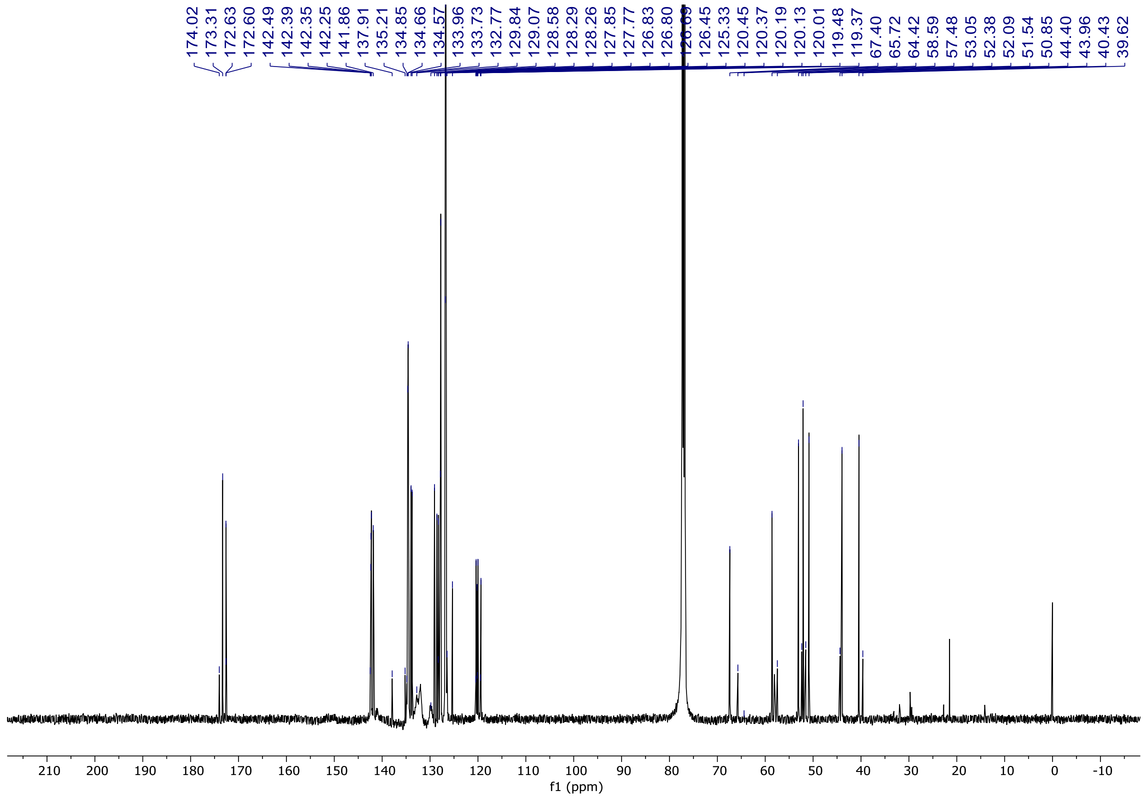

Figure S5. ^{13}C NMR spectrum of compound **3** in $CDCl_3$.

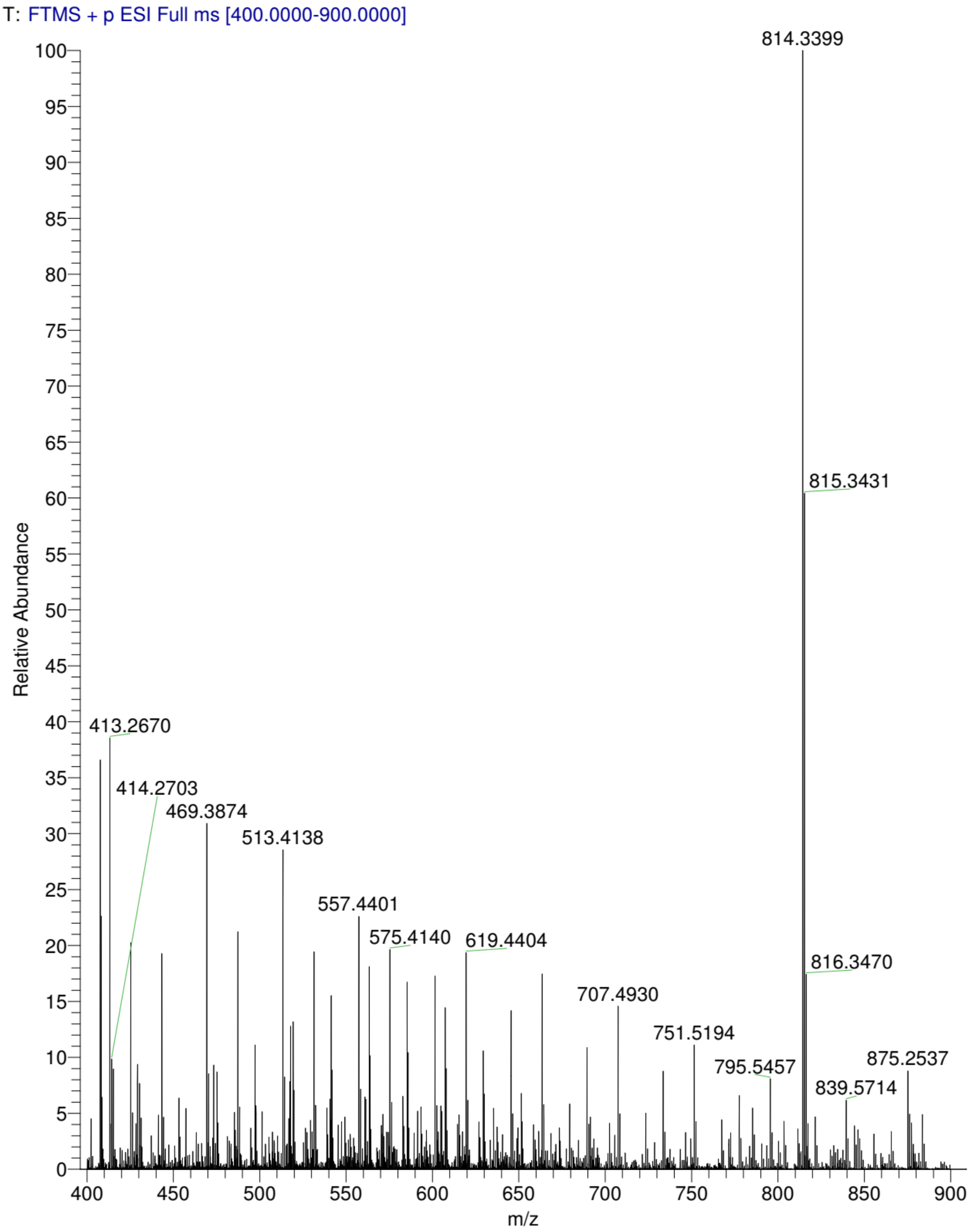

Figure S6. HRMS-ESI(+) spectrum of compound **3**.

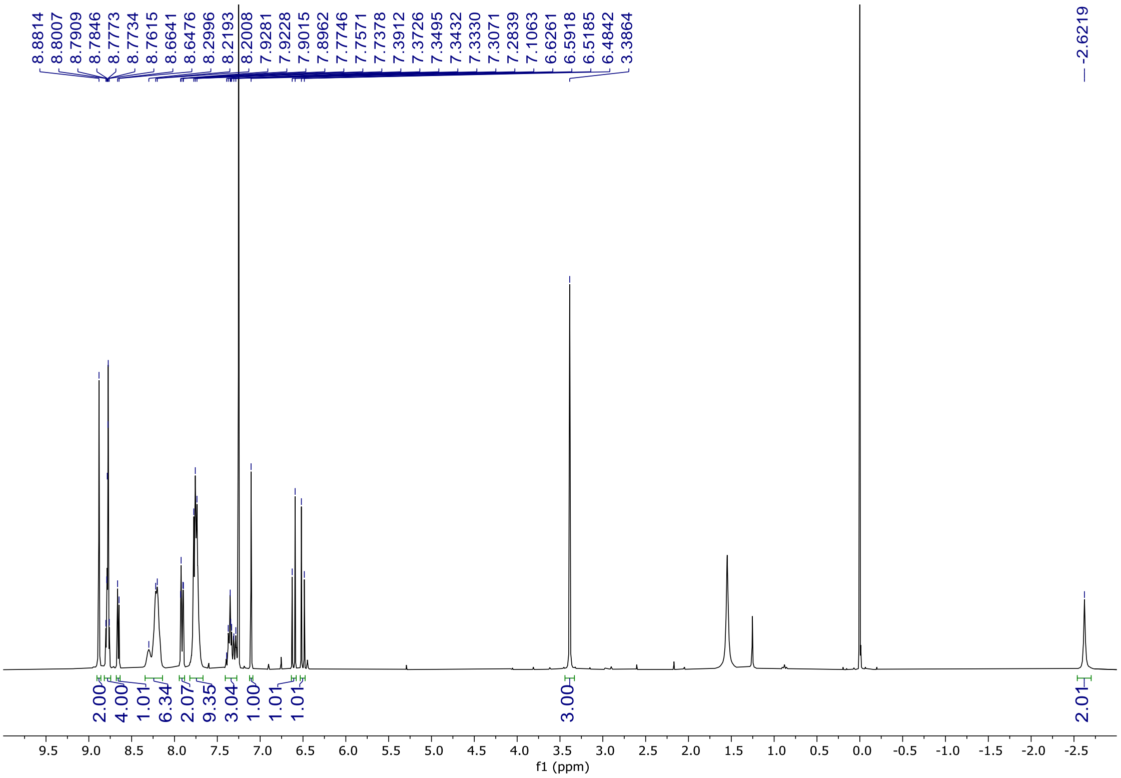

Figure S7. ^{1}H NMR spectrum of compound **4** in $CDCl_3$.

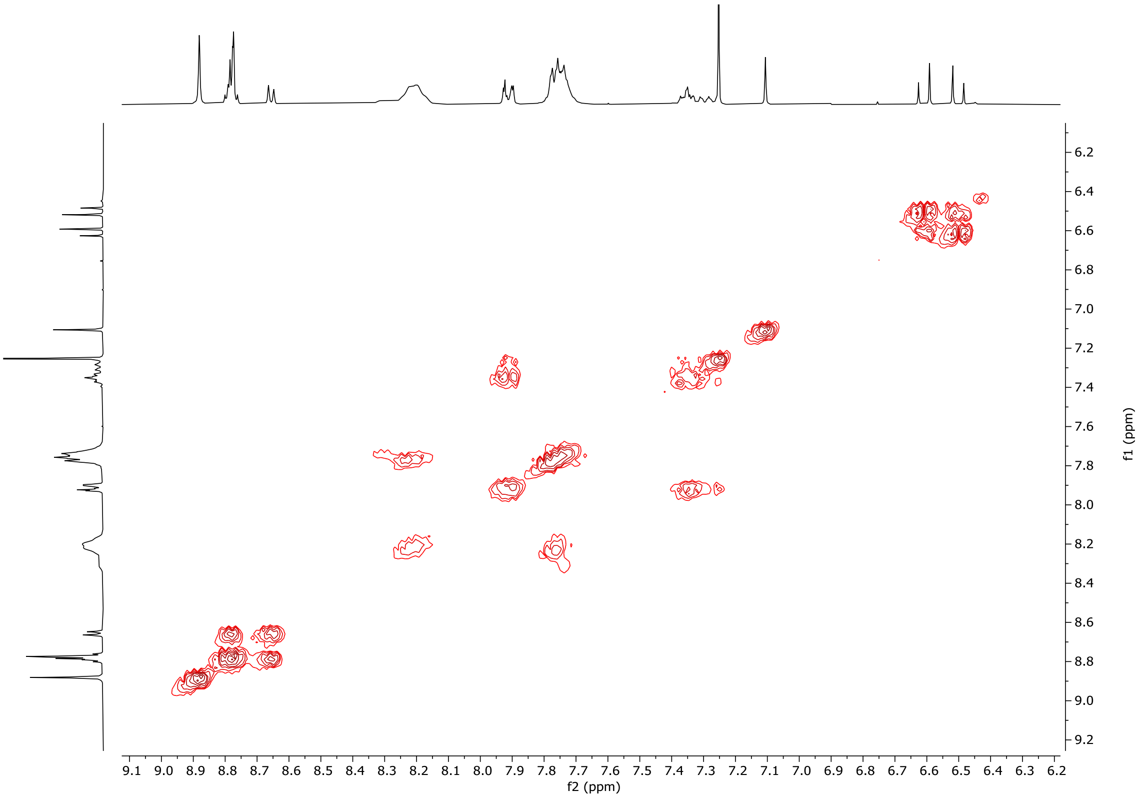

Figure S8. Partial COSY $^{1}H/^{1}H$ NMR spectrum of compound **4** in $CDCl_3$.

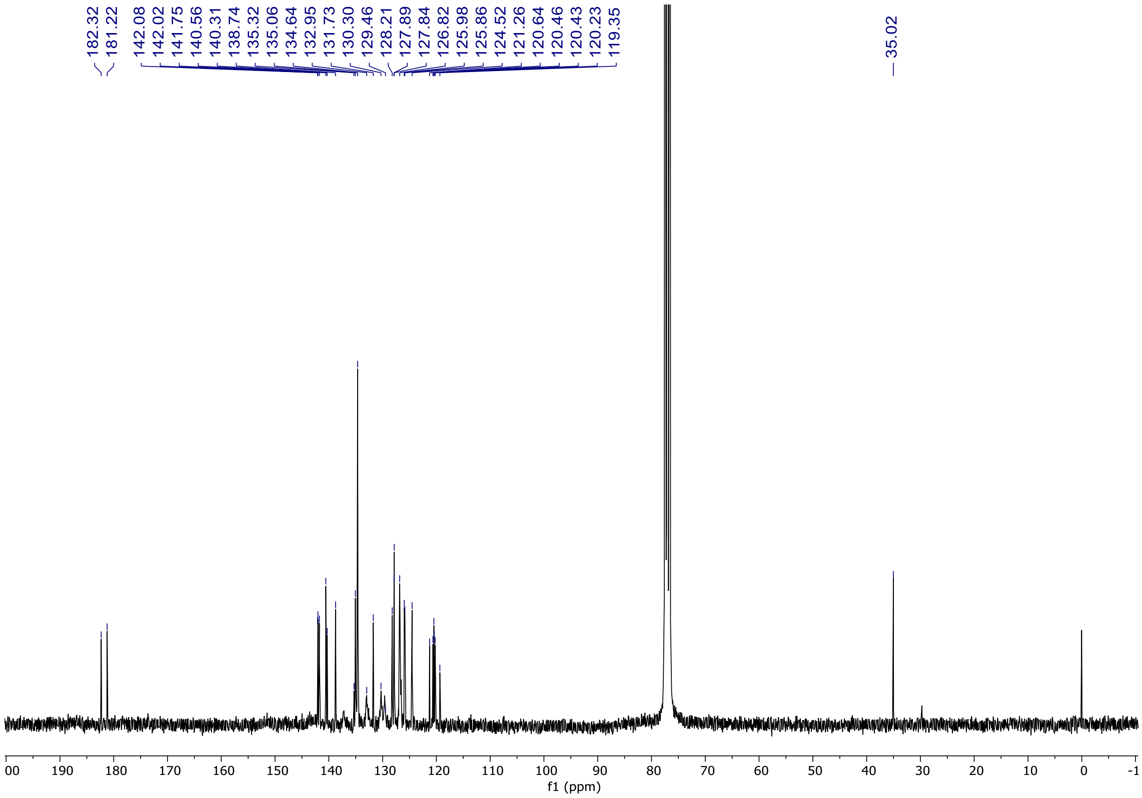

Figure S9. ^{13}C NMR spectrum of compound **4** in $CDCl_3$.

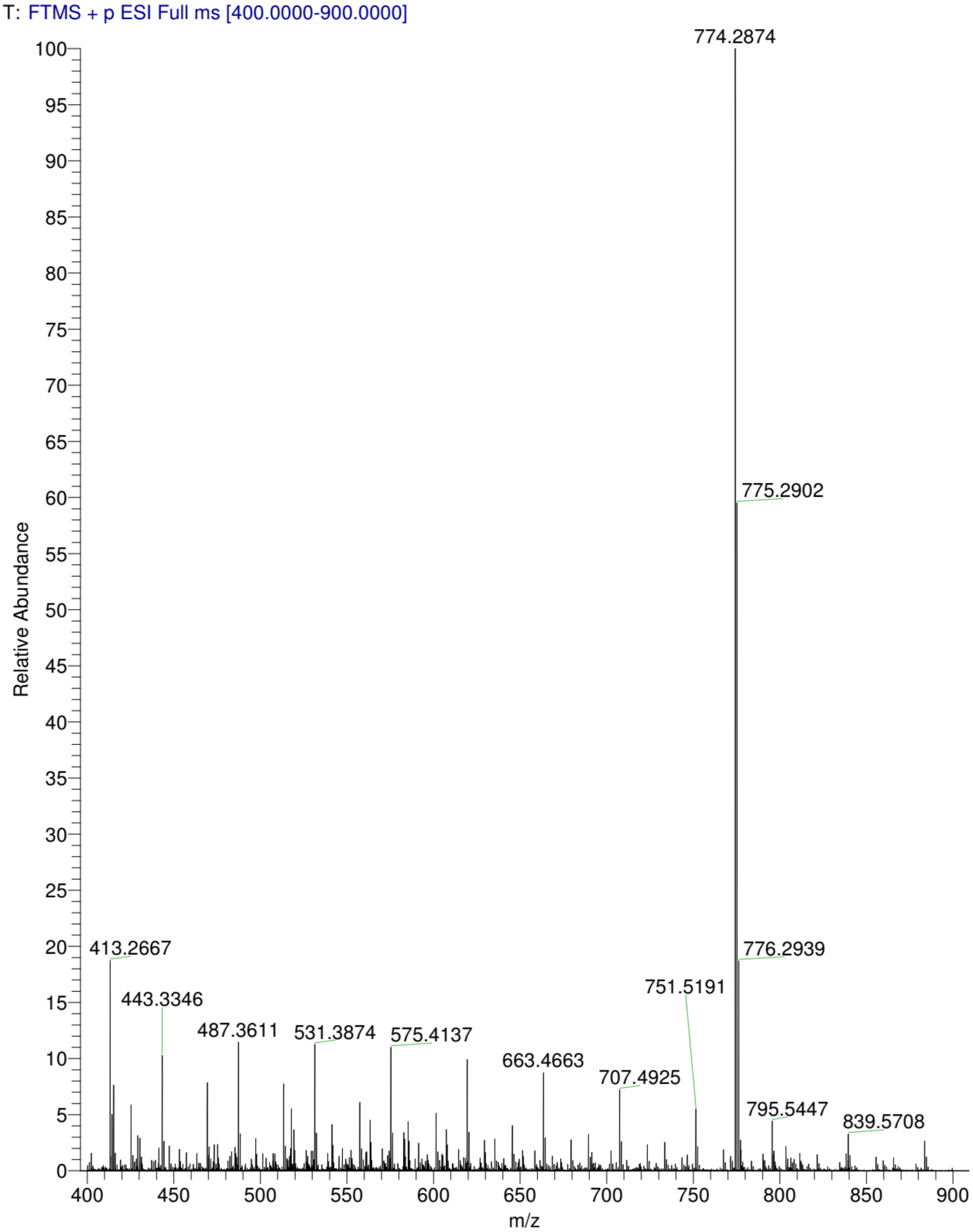

Figure S10. HRMS-ESI(+) spectrum of compound **4**.

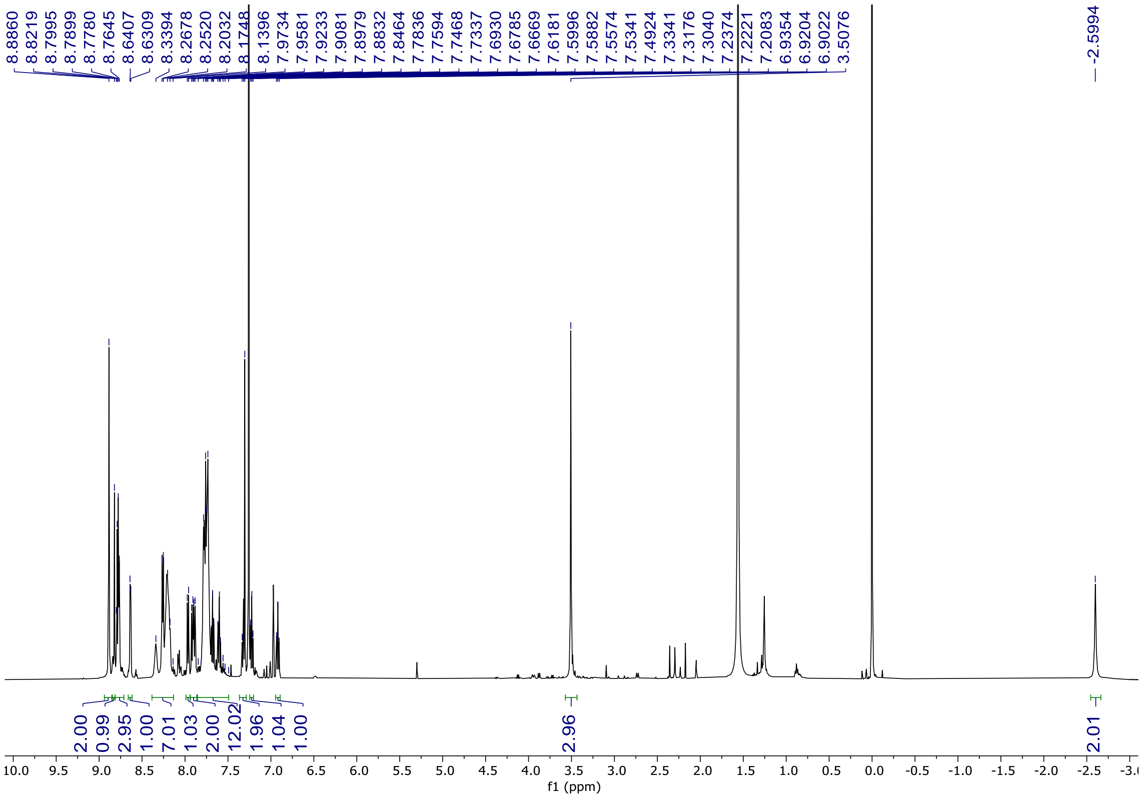

Figure S11. ^{1}H NMR spectrum of compound **5a** in $CDCl_3$.

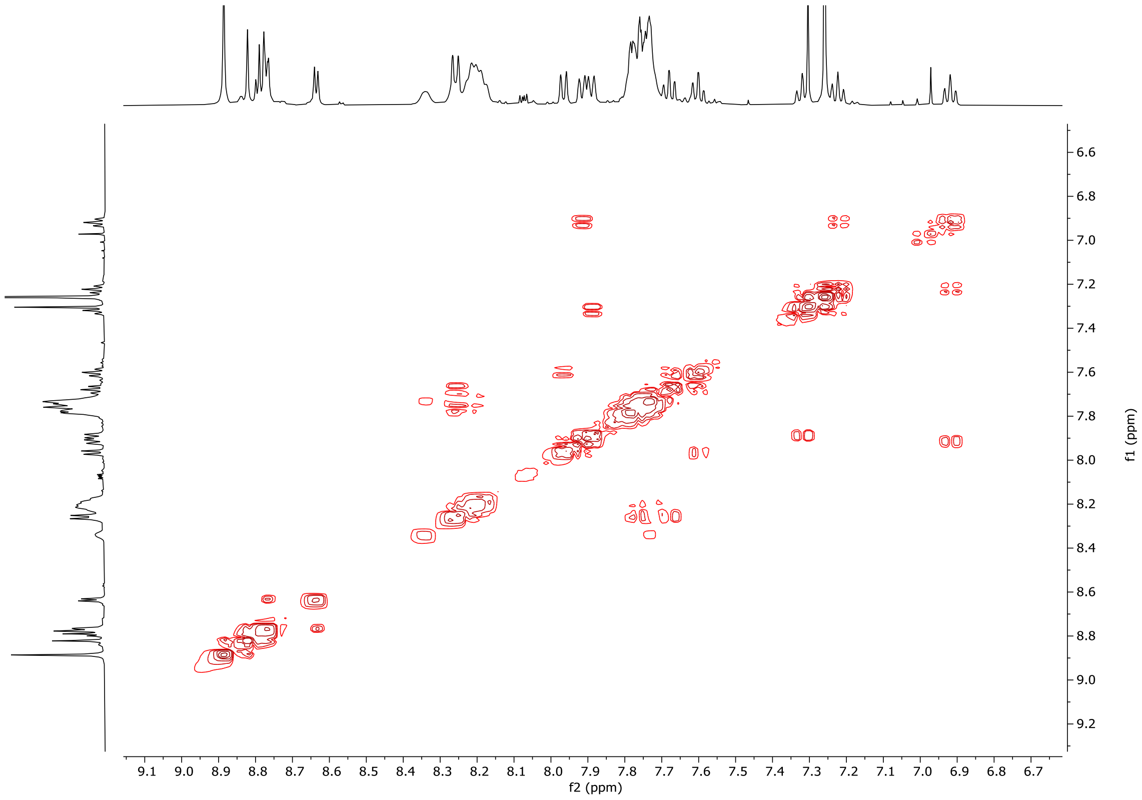

Figure S12. Partial COSY $^{1}H/^{1}H$ NMR spectrum of compound **5a** in $CDCl_3$.

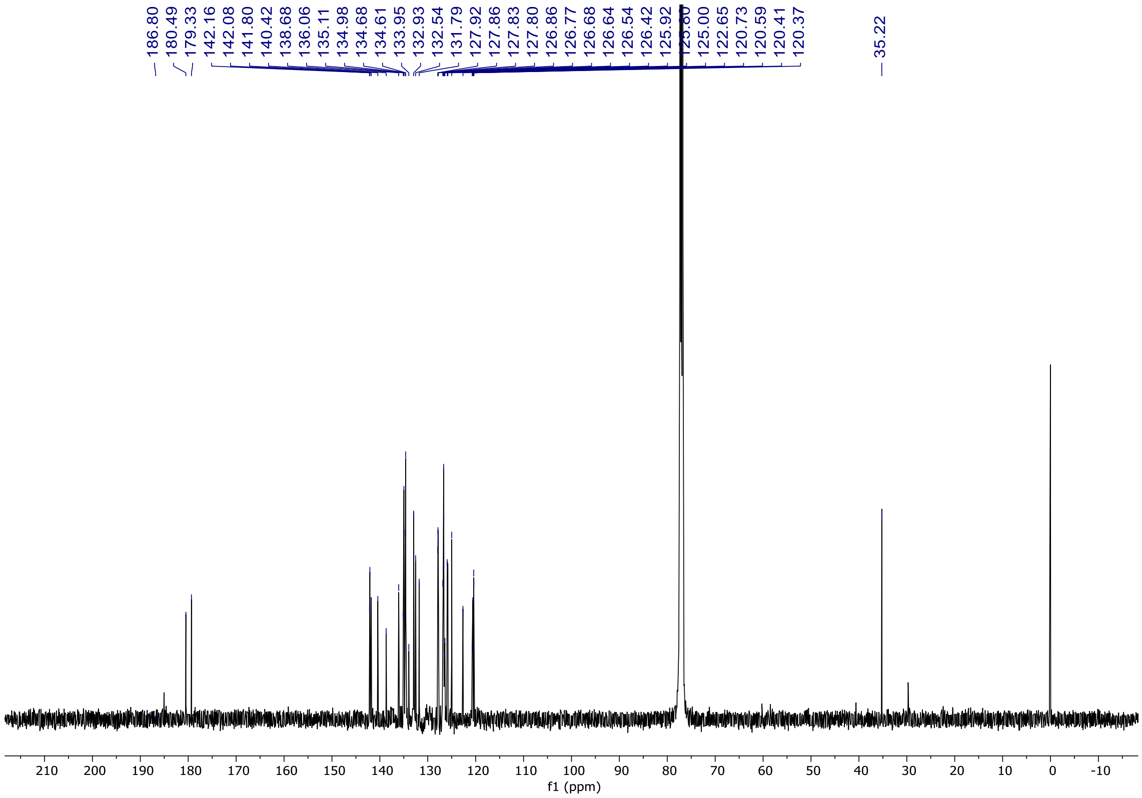

Figure S13. ^{13}C NMR spectrum of compound **5a** in $CDCl_3$.

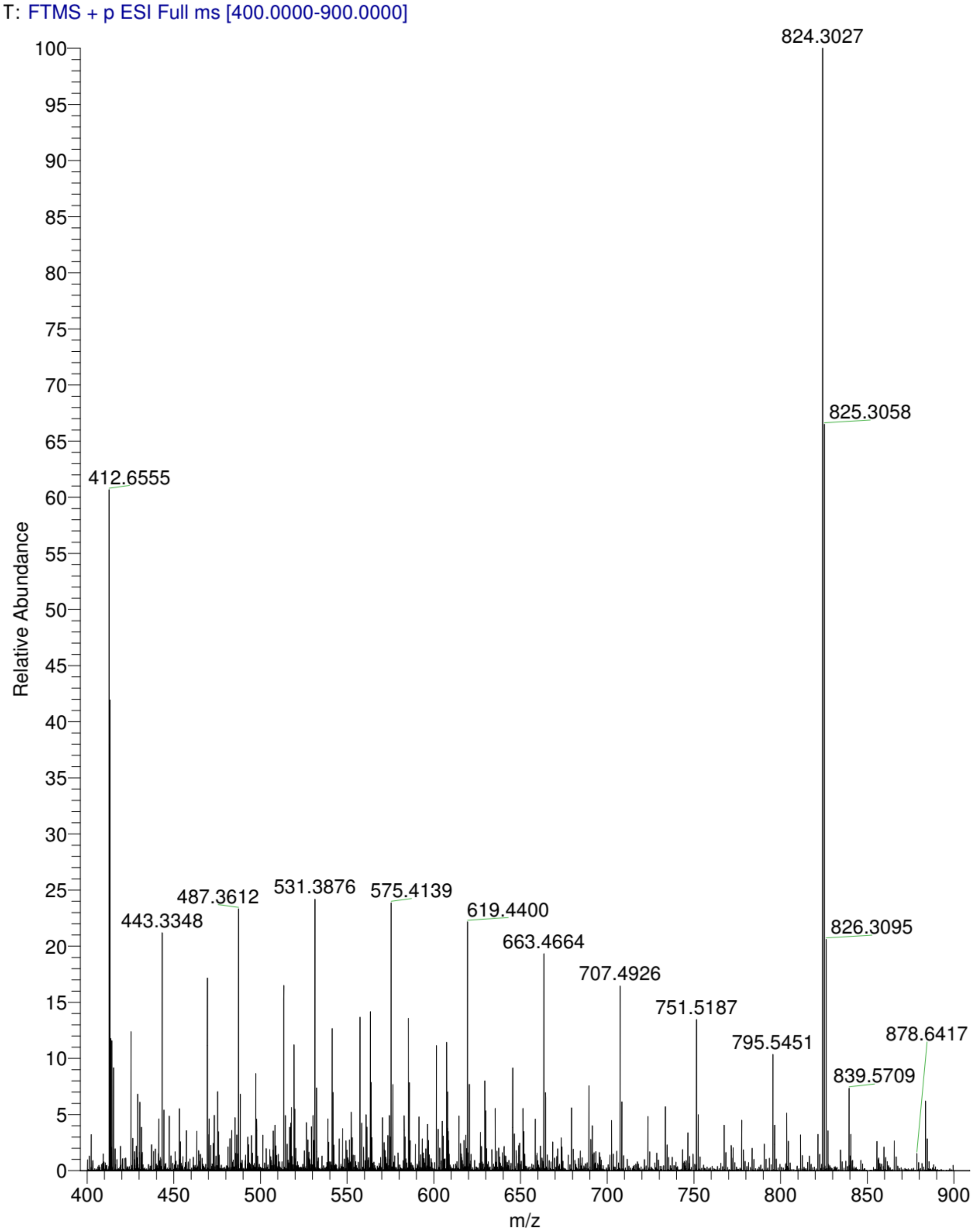

Figure S14. HRMS-ESI(+) spectrum of compound **5a**.

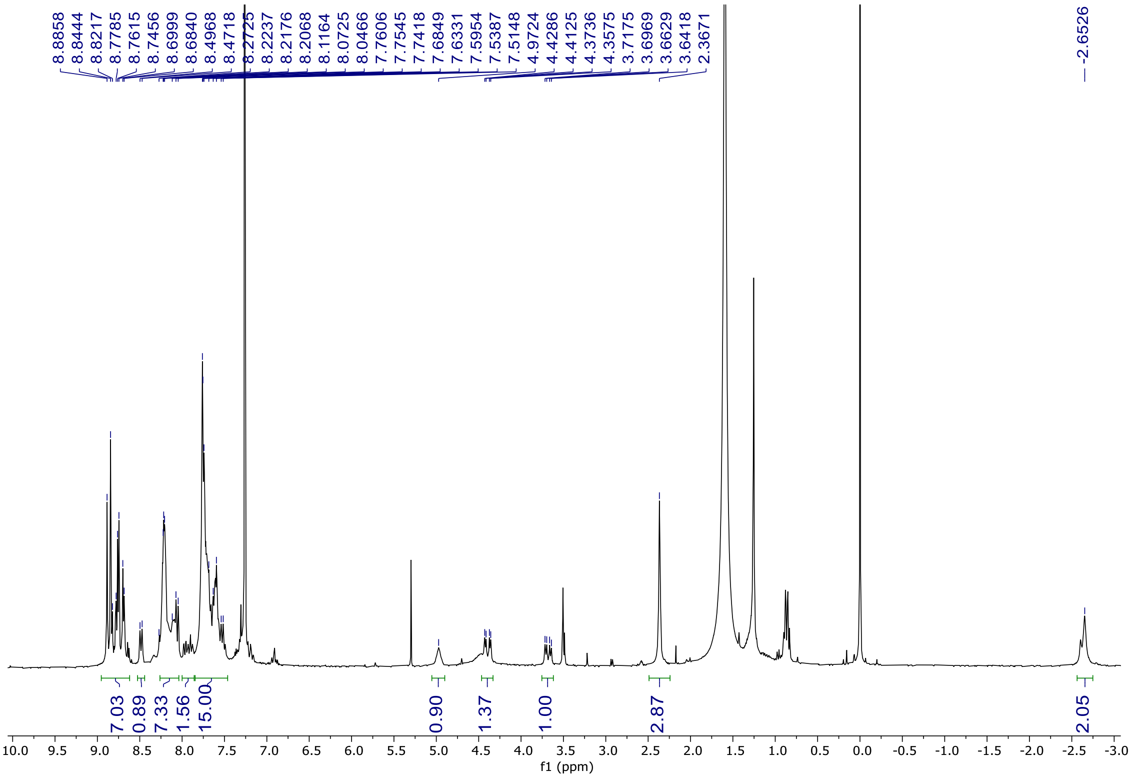

Figure S15. ^{1}H NMR spectrum of compound **5b** in $CDCl_3$.

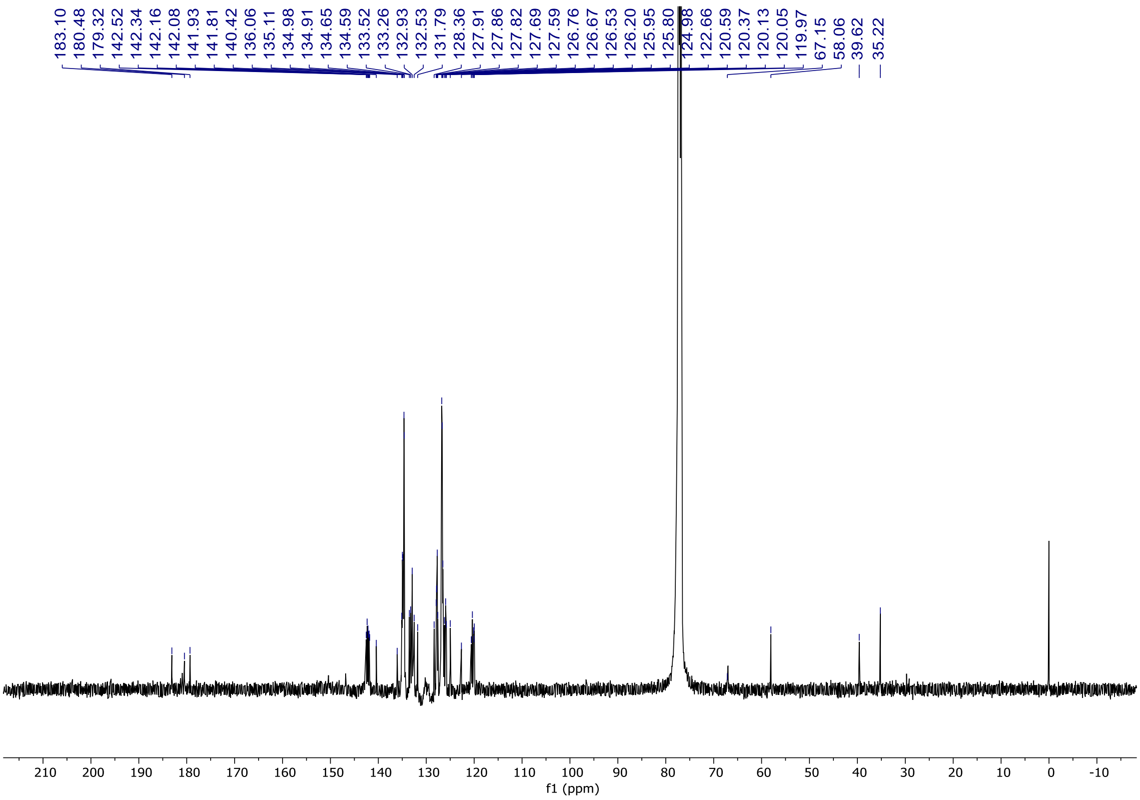

Figure S16. ^{13}C NMR spectrum of compound **5b** in $CDCl_3$.

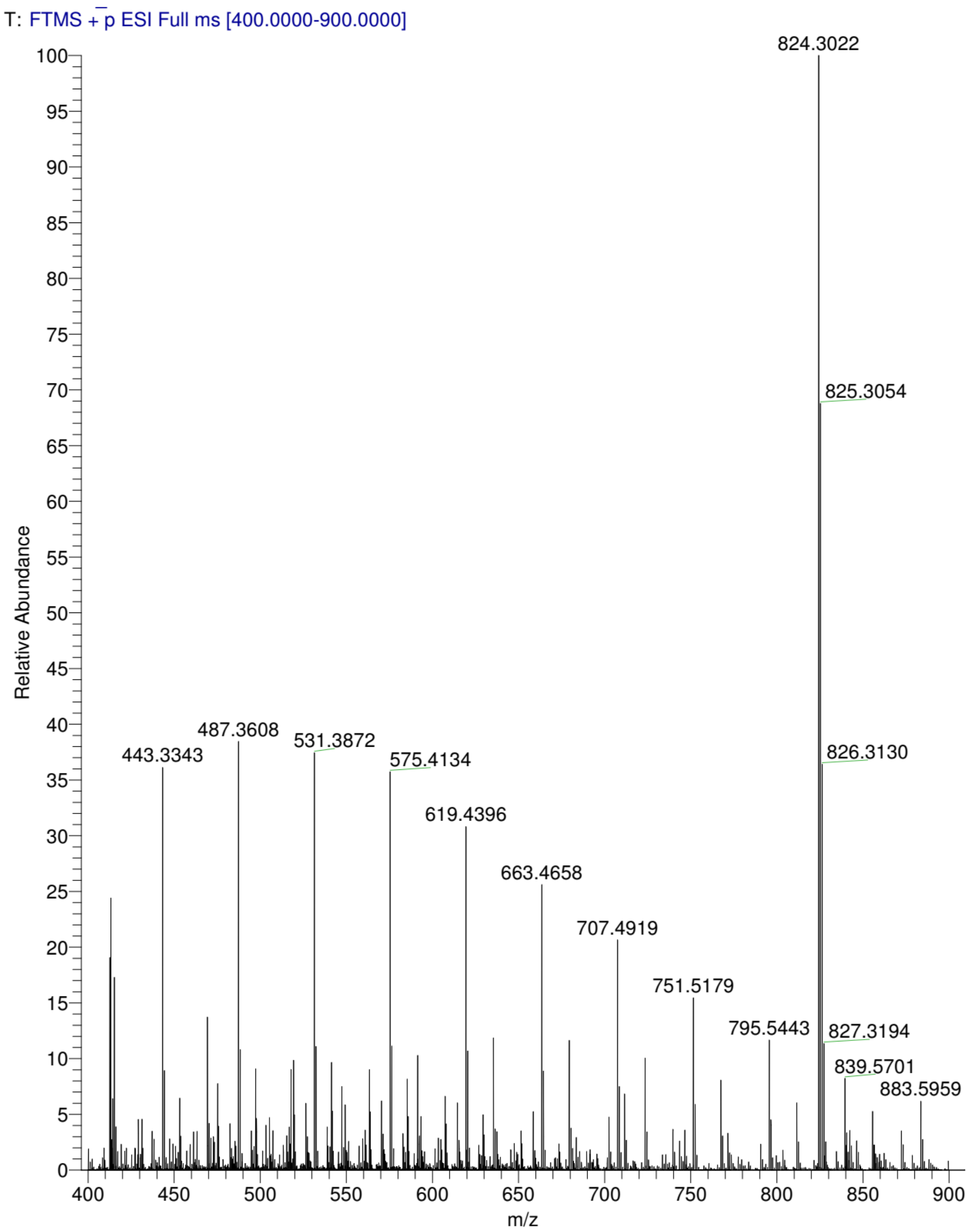

Figure S17. MS-ESI(+) spectrum of compound **5b**.

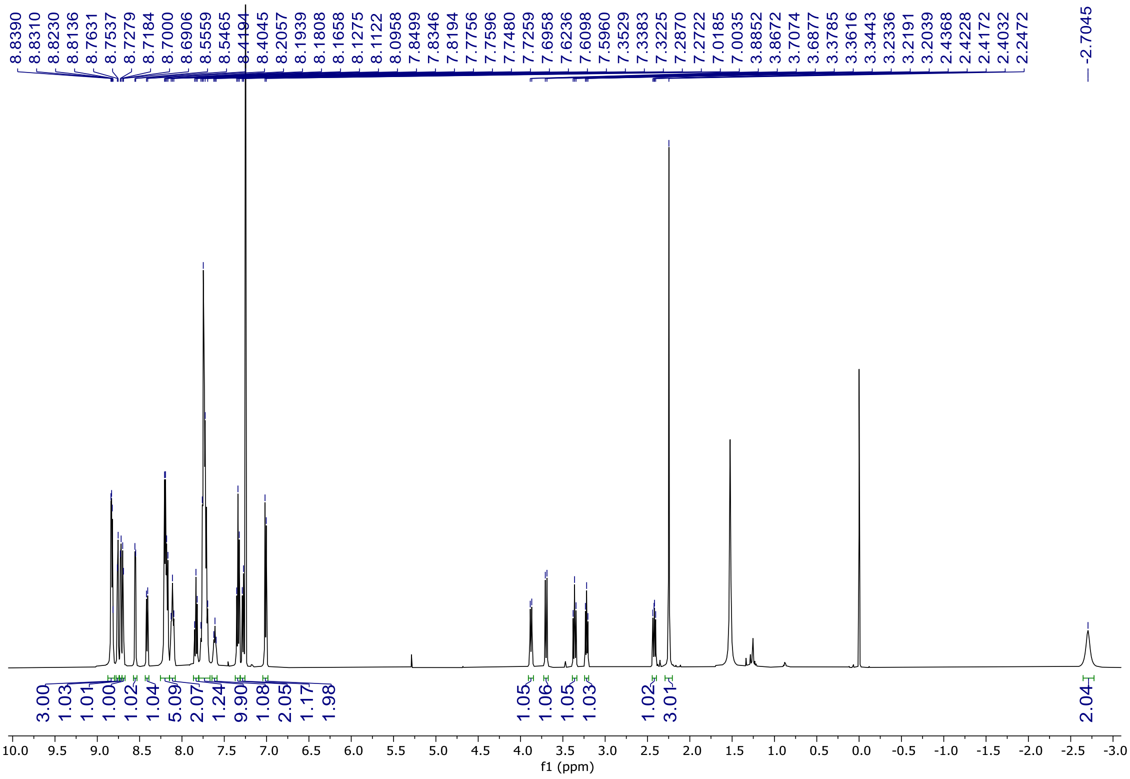

Figure S18. ^{1}H NMR spectrum of compound **6a** in $CDCl_3$.

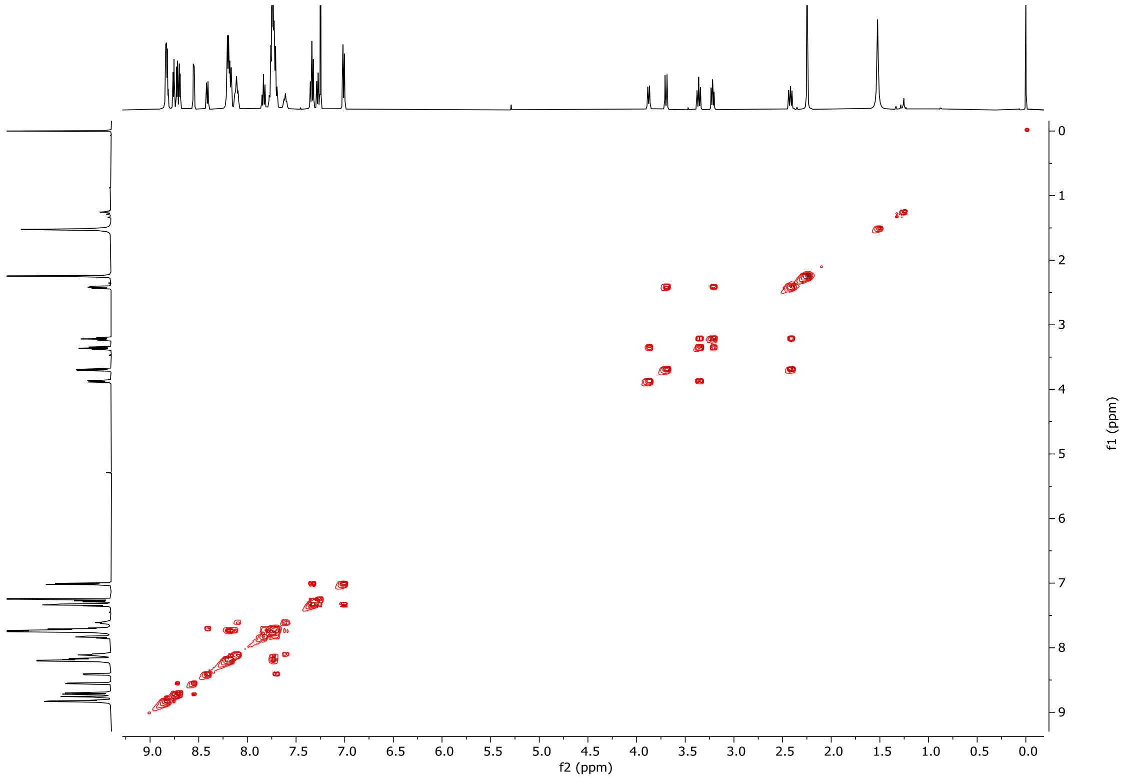

Figure S19. Partial COSY $^{1}H/^{1}H$ NMR spectrum of compound **6a** in $CDCl_3$.

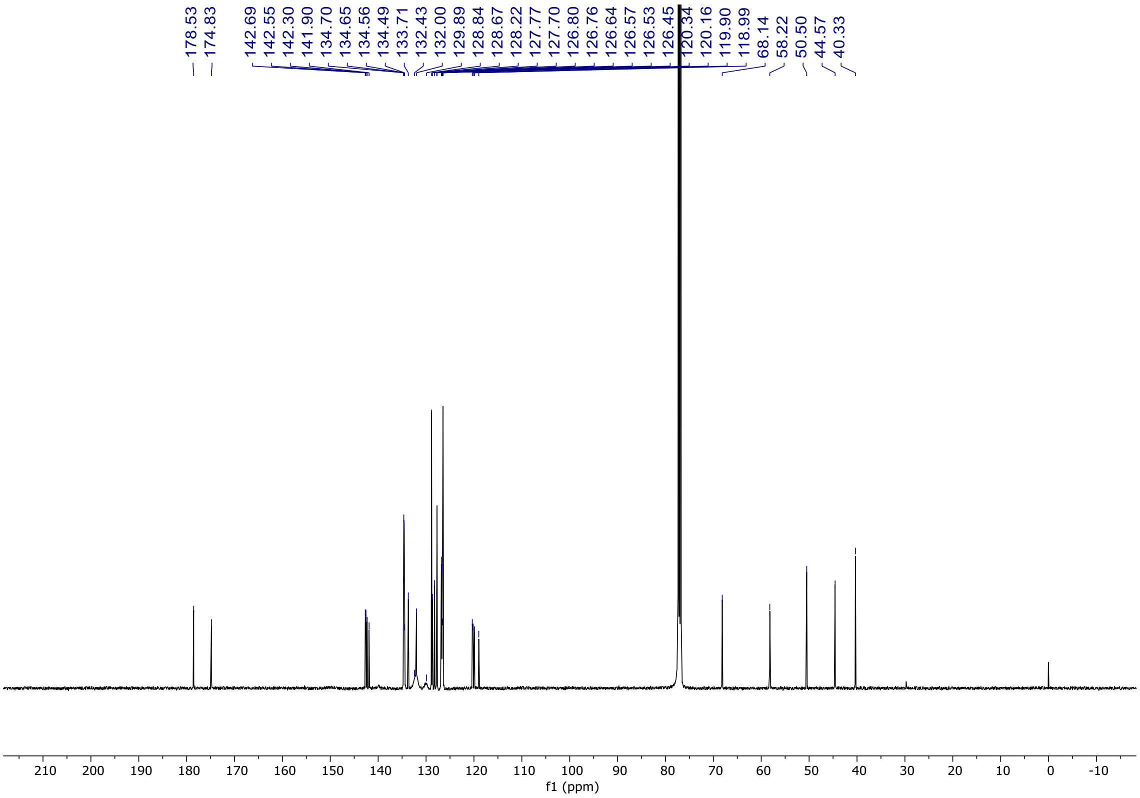

Figure S20. ^{13}C NMR spectrum of compound **6a** in $CDCl_3$.

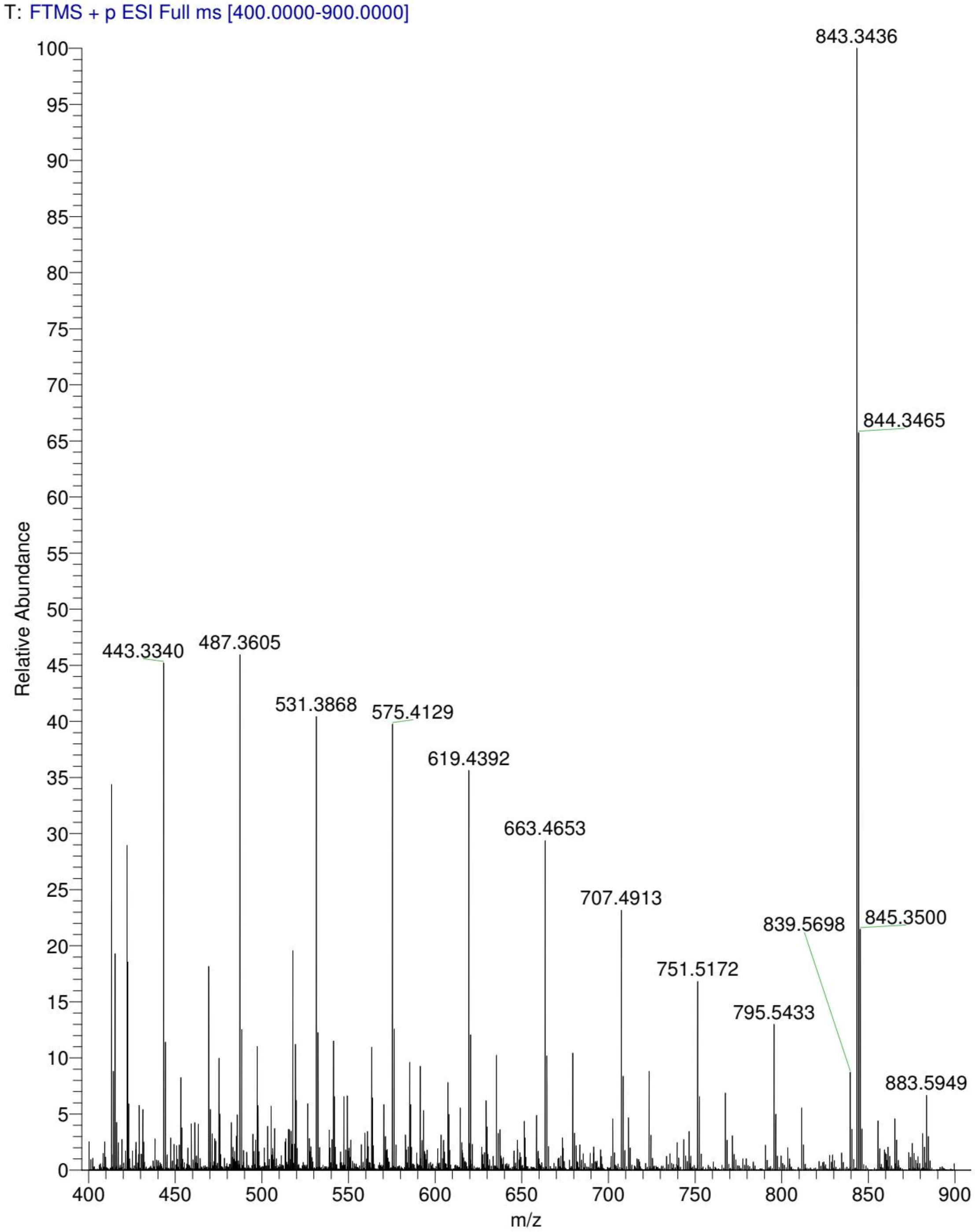

Figure S21. HRMS-ESI(+) spectrum of compound **6a**.

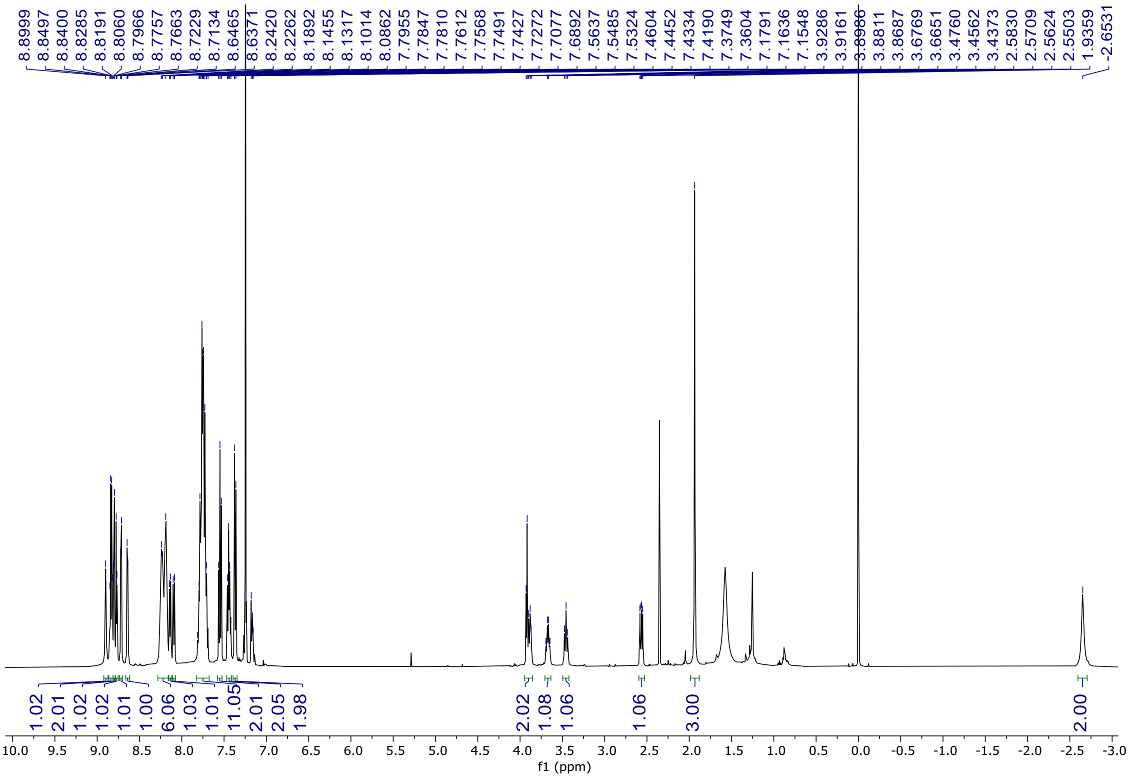

Figure S22. ^{1}H NMR spectrum of compound **6b** in $CDCl_3$.

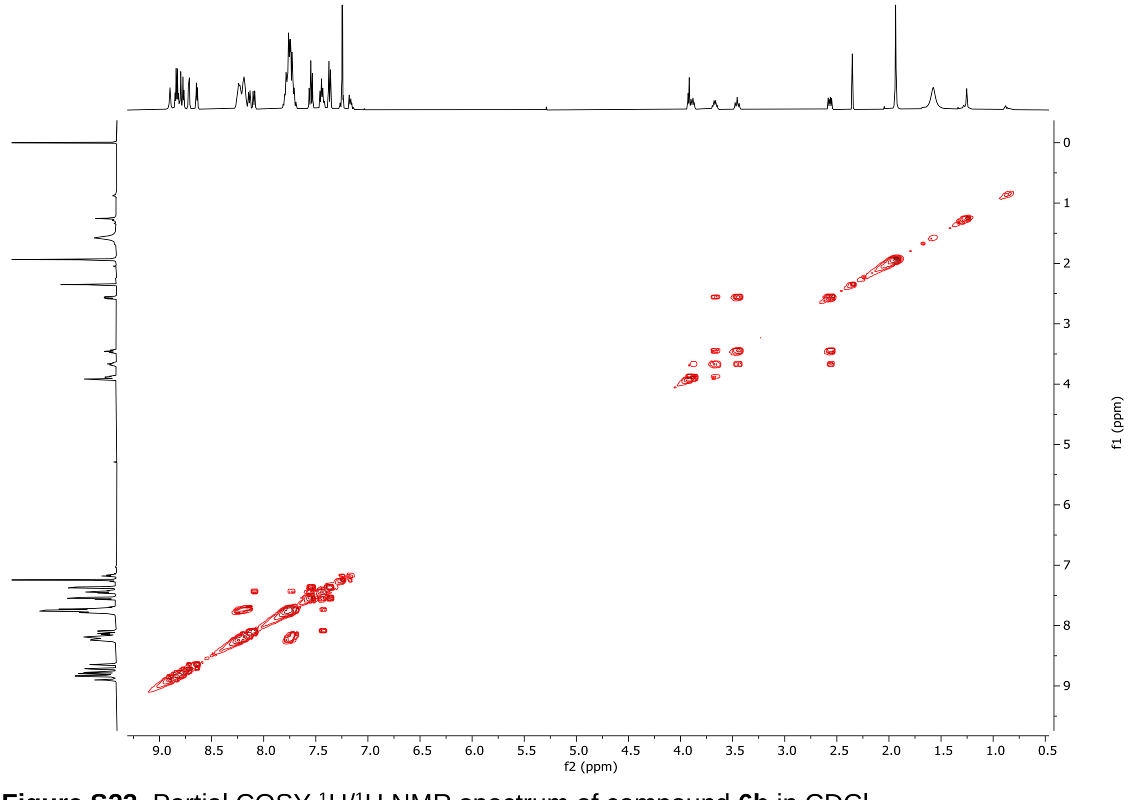

Figure S23. Partial COSY $^{1}H/^{1}H$ NMR spectrum of compound **6b** in $CDCl_3$.

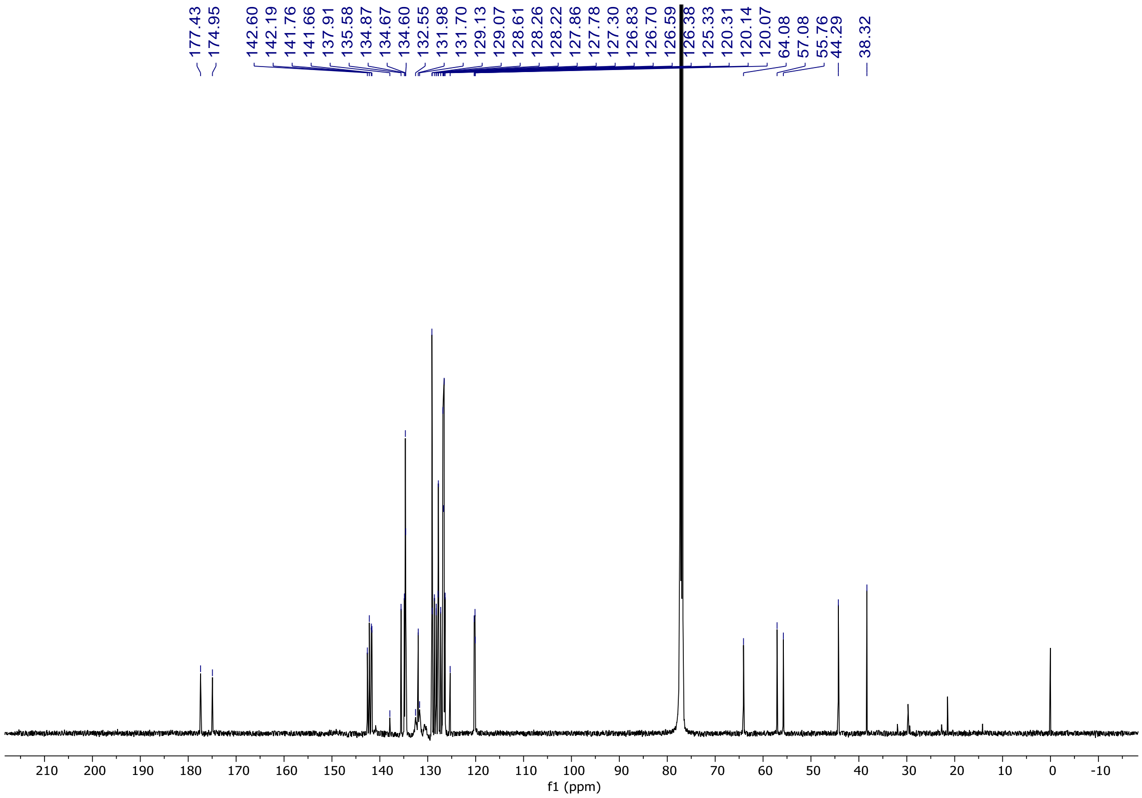

Figure S24. ^{13}C NMR spectrum of compound **6b** in $CDCl_3$.

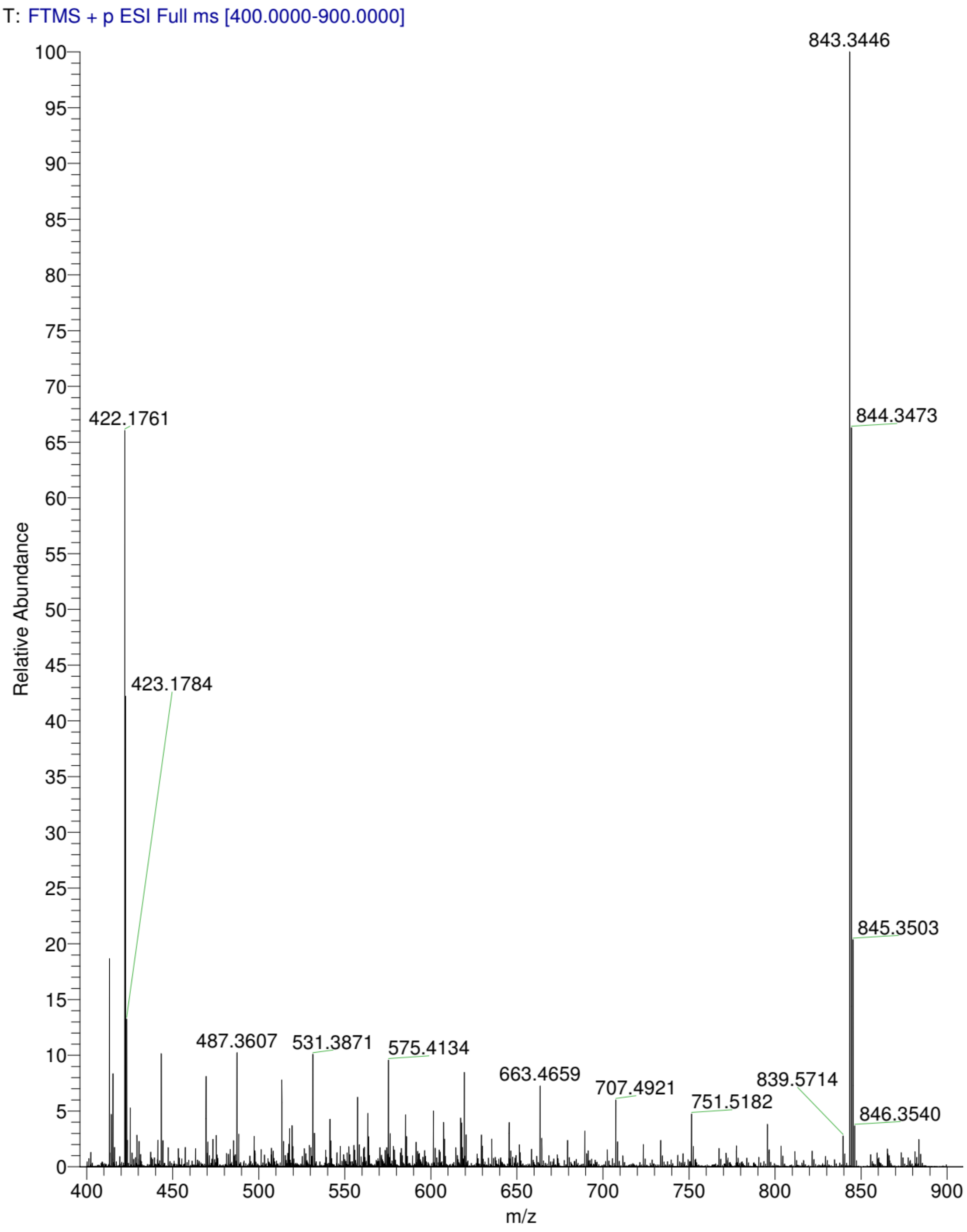

Figure S25. HRMS-ESI(+) spectrum of compound **6b**.

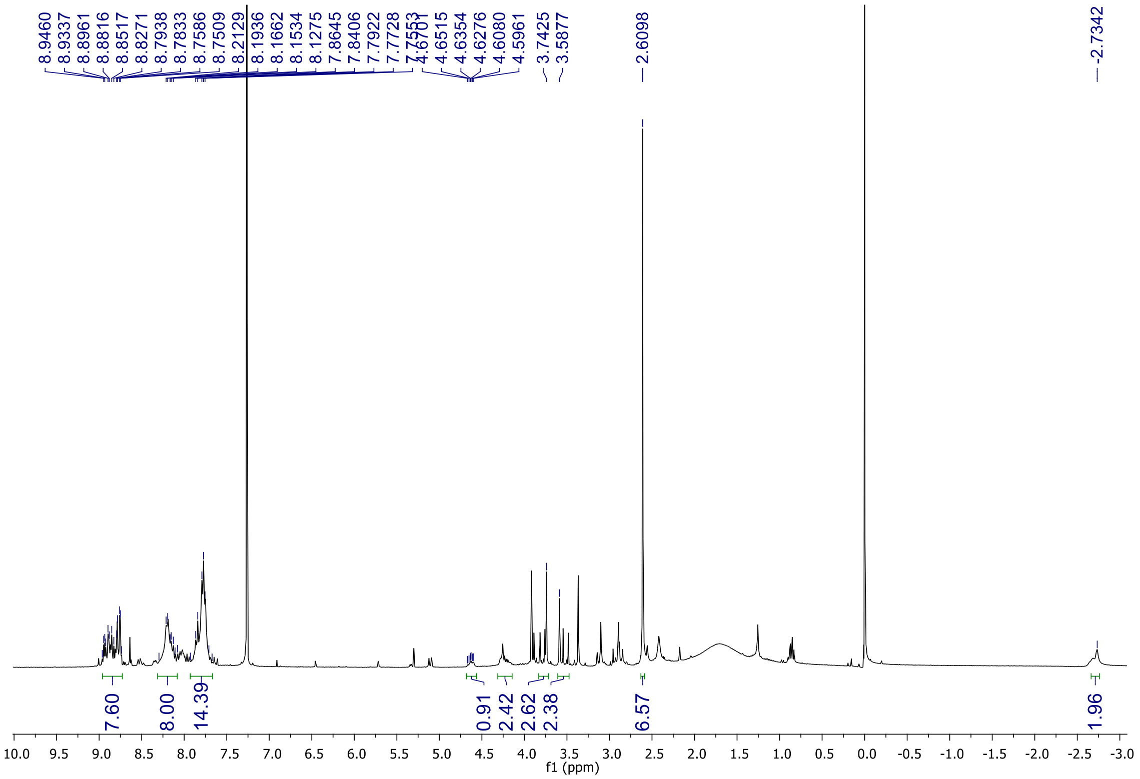

Figure S26. ^{1}H NMR spectrum of compound **7a** in $CDCl_3$.

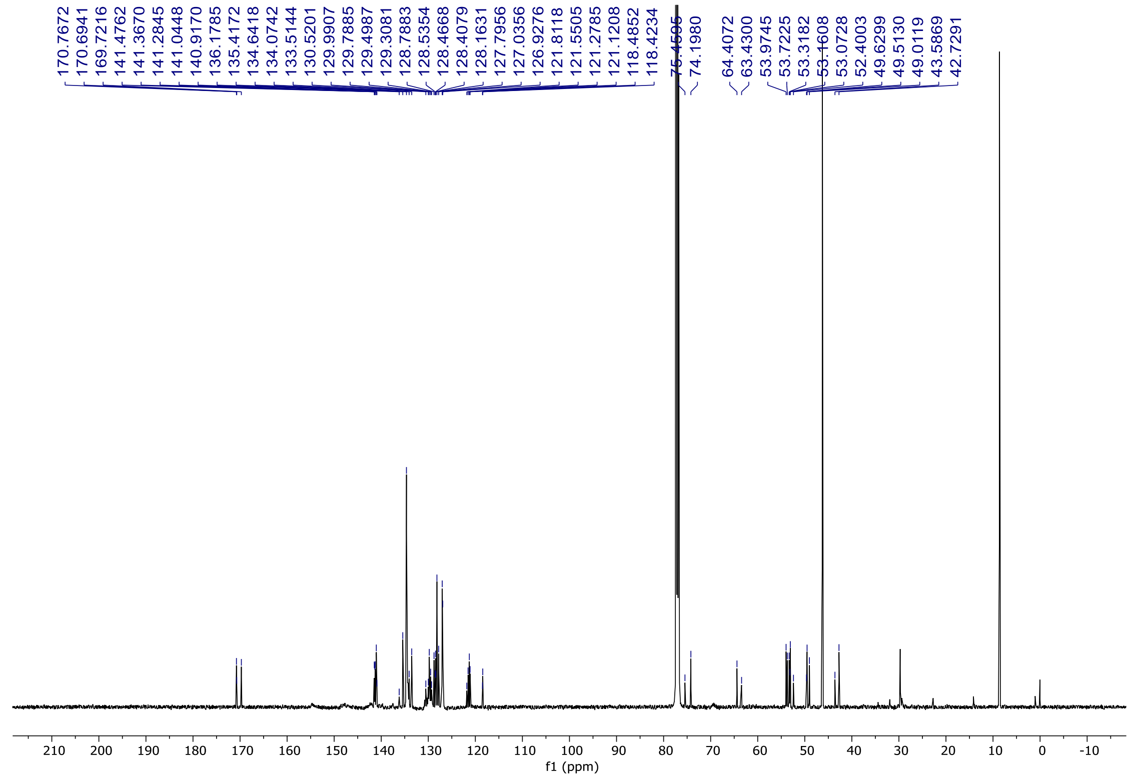

Figure S27. ^{13}C NMR spectrum of compound **7a** in $CDCl_3$.

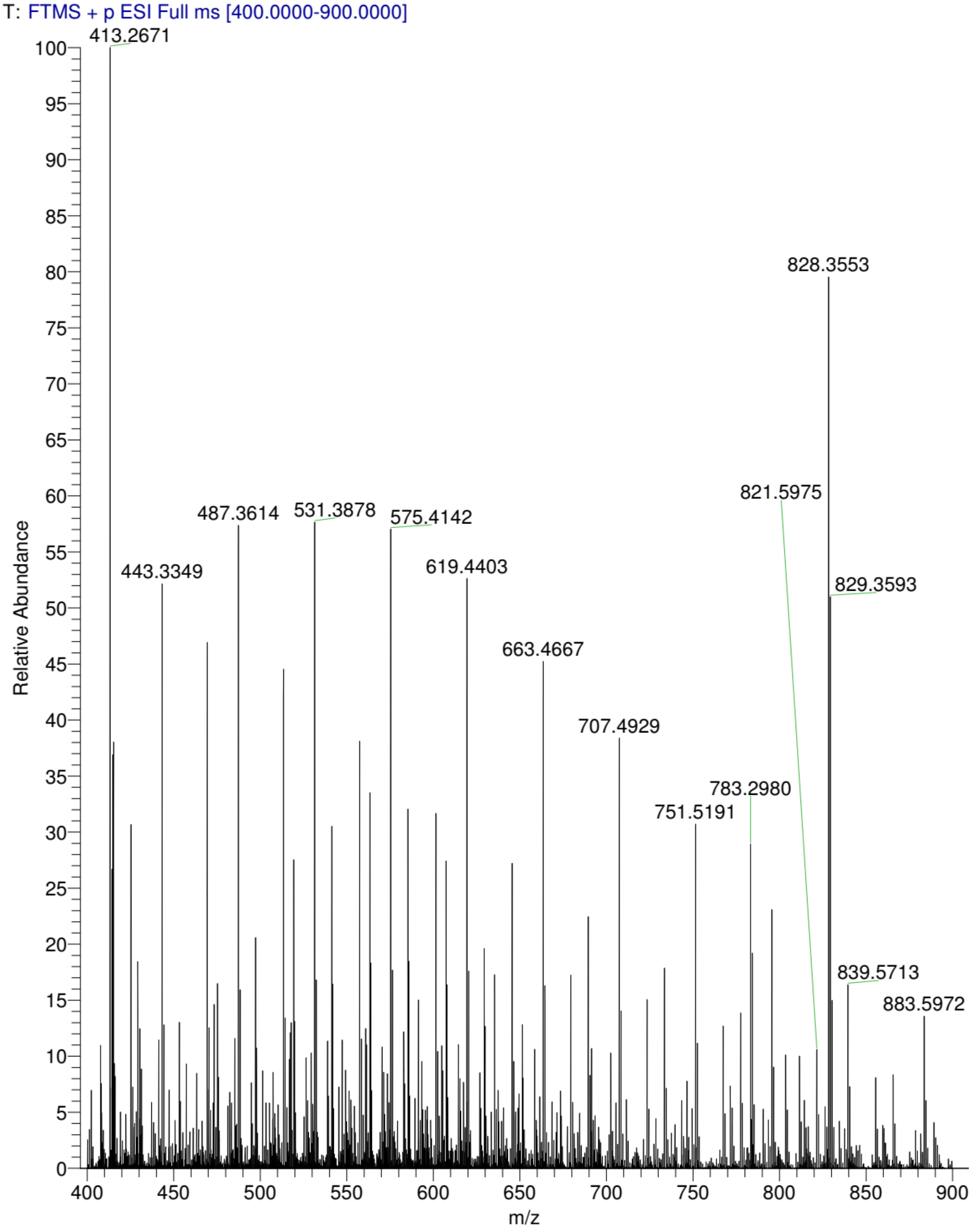

Figure S28. HRMS-ESI(+) spectrum of compound **7a**.

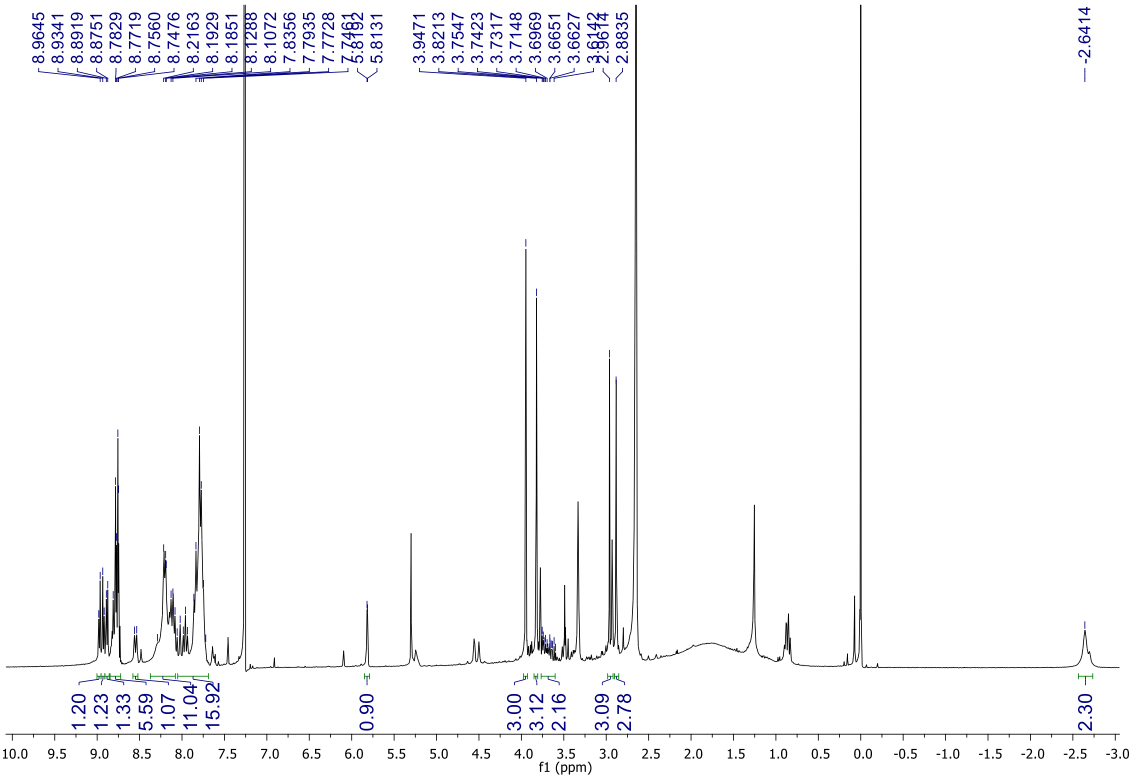

Figure S29. ^{1}H NMR spectrum of compound **7b** in $CDCl_3$.

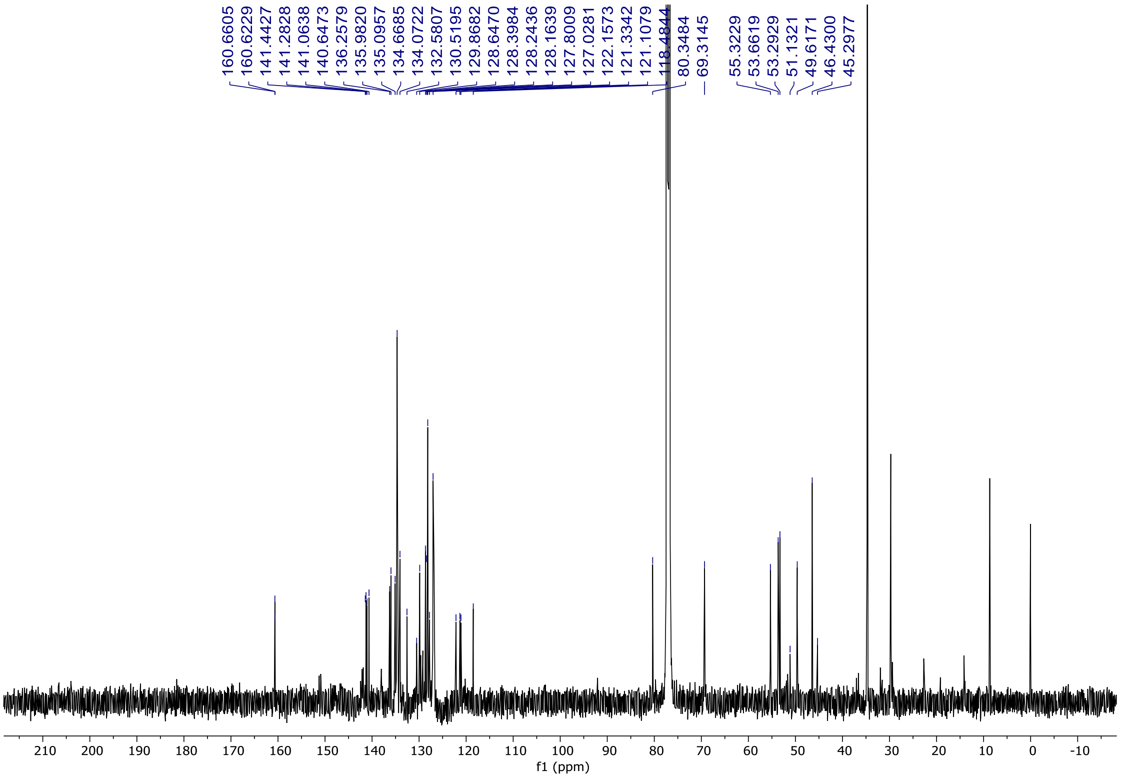

Figure S30. ^{13}C NMR spectrum of compound **7b** in $CDCl_3$.

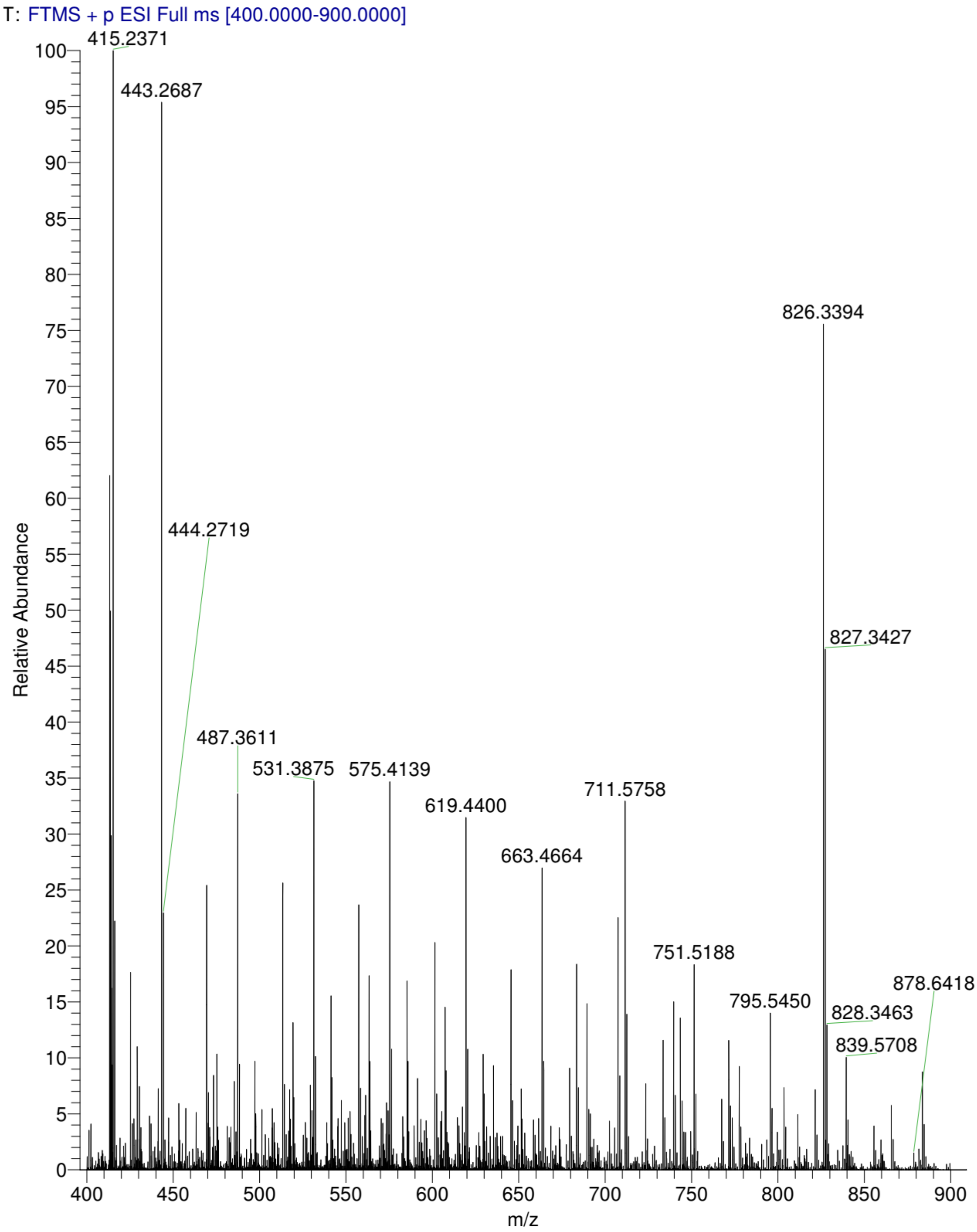

Figure S31. HRMS-ESI(+) spectrum of compound **7b**.

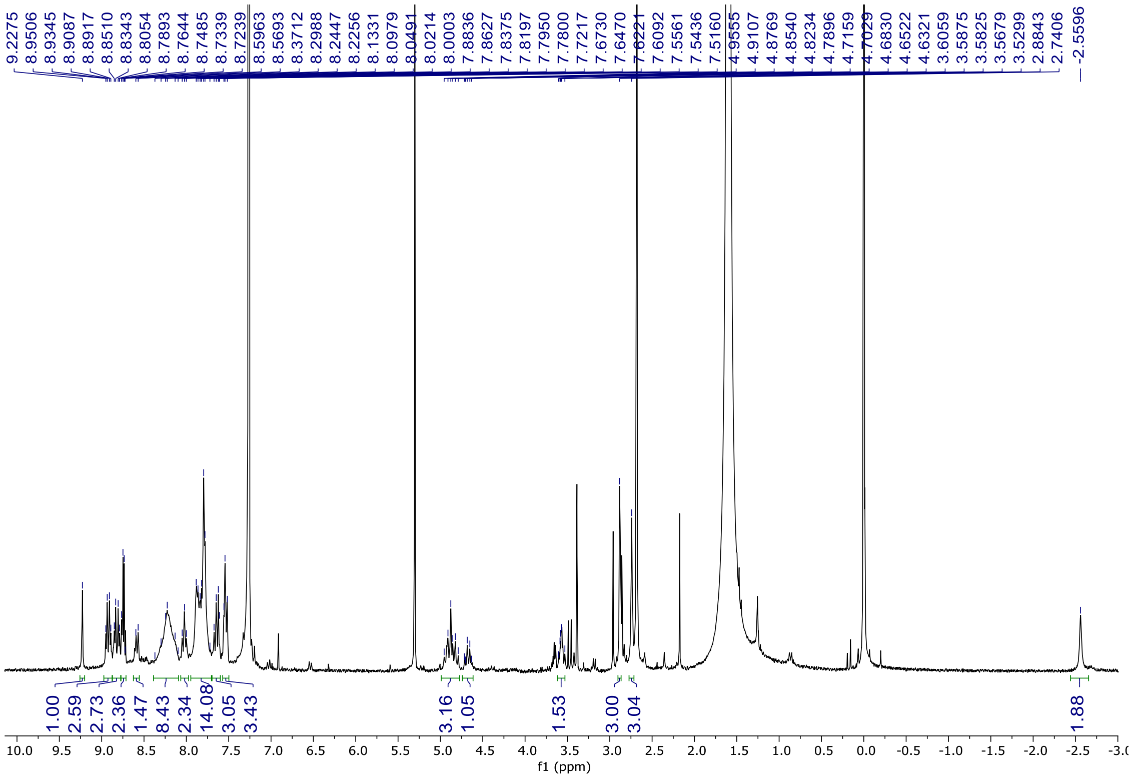

Figure S32. ^{1}H NMR spectrum of compound **7c** in $CDCl_3$.

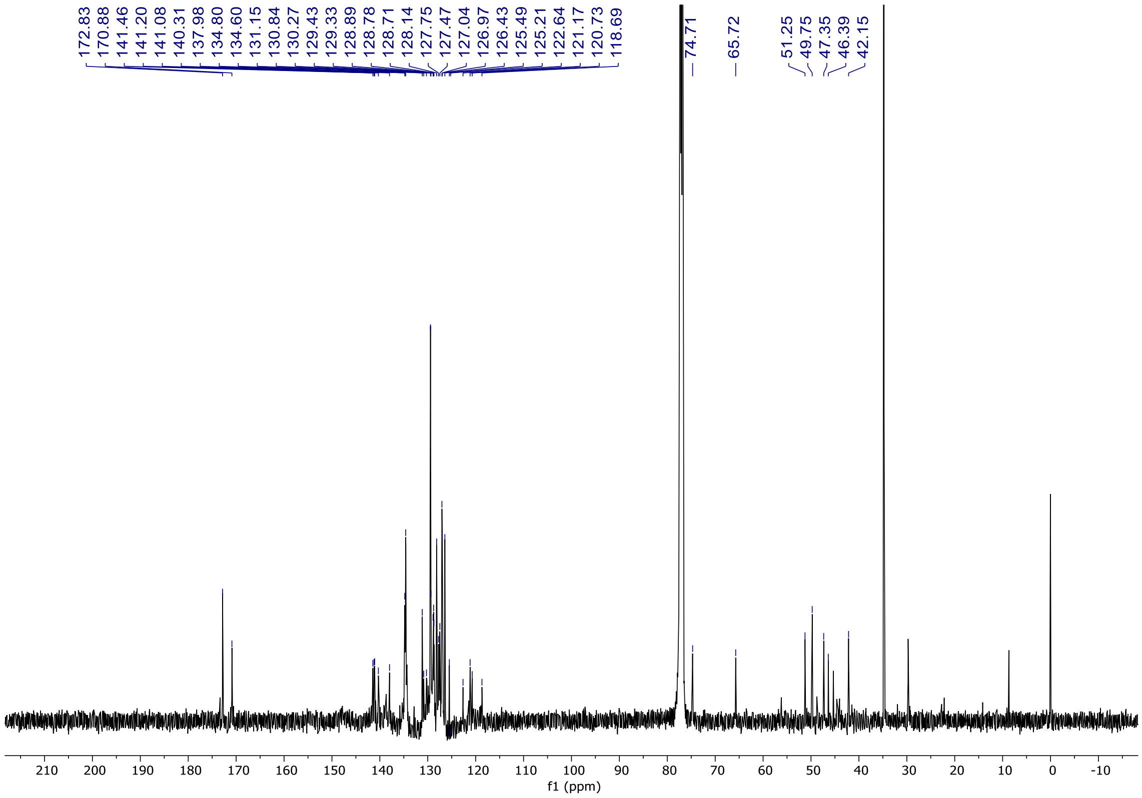

Figure S33. ^{13}C NMR spectrum of compound **7c** in $CDCl_3$.

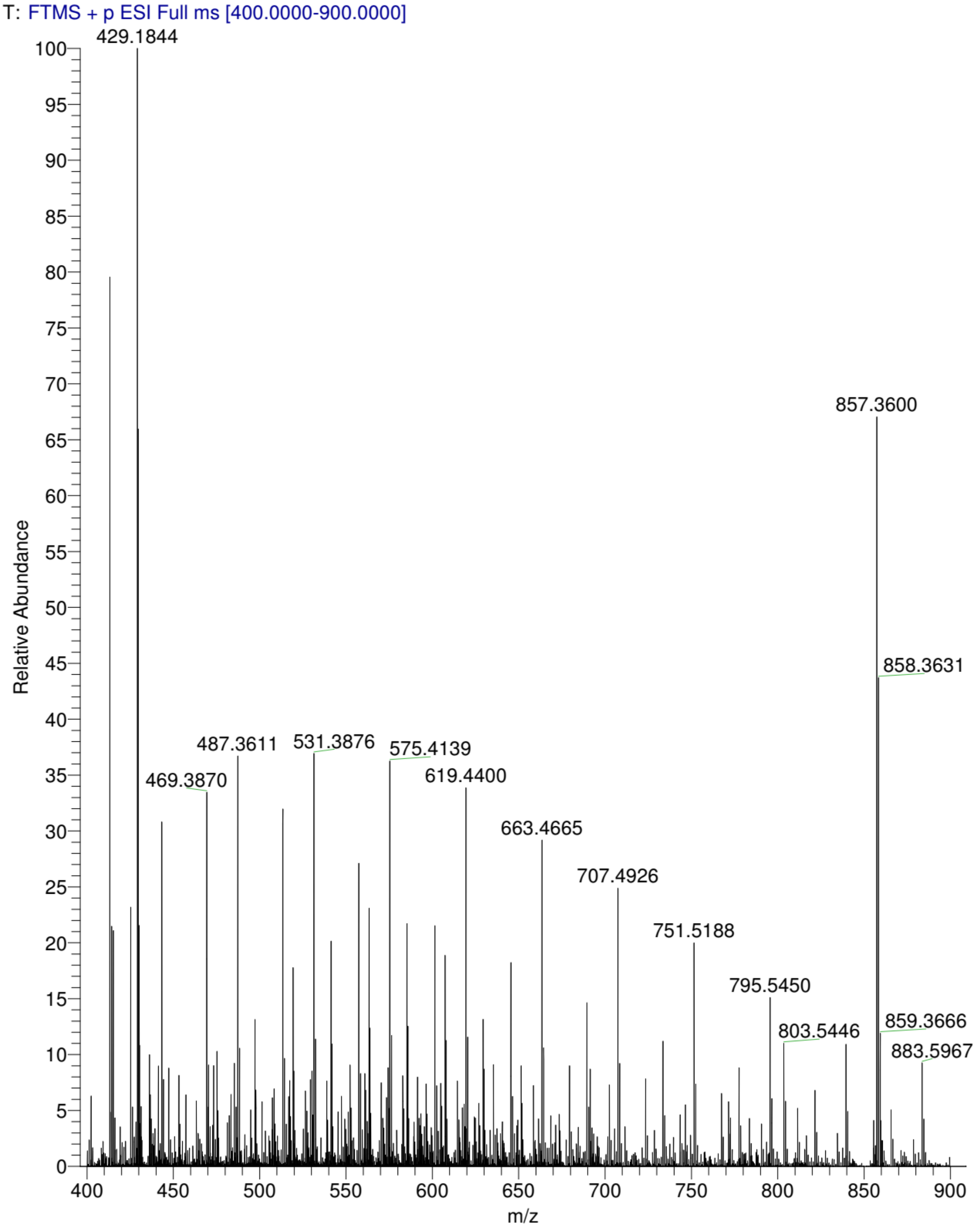

Figure S34. HRMS-ESI(+) spectrum of compound **7c**.

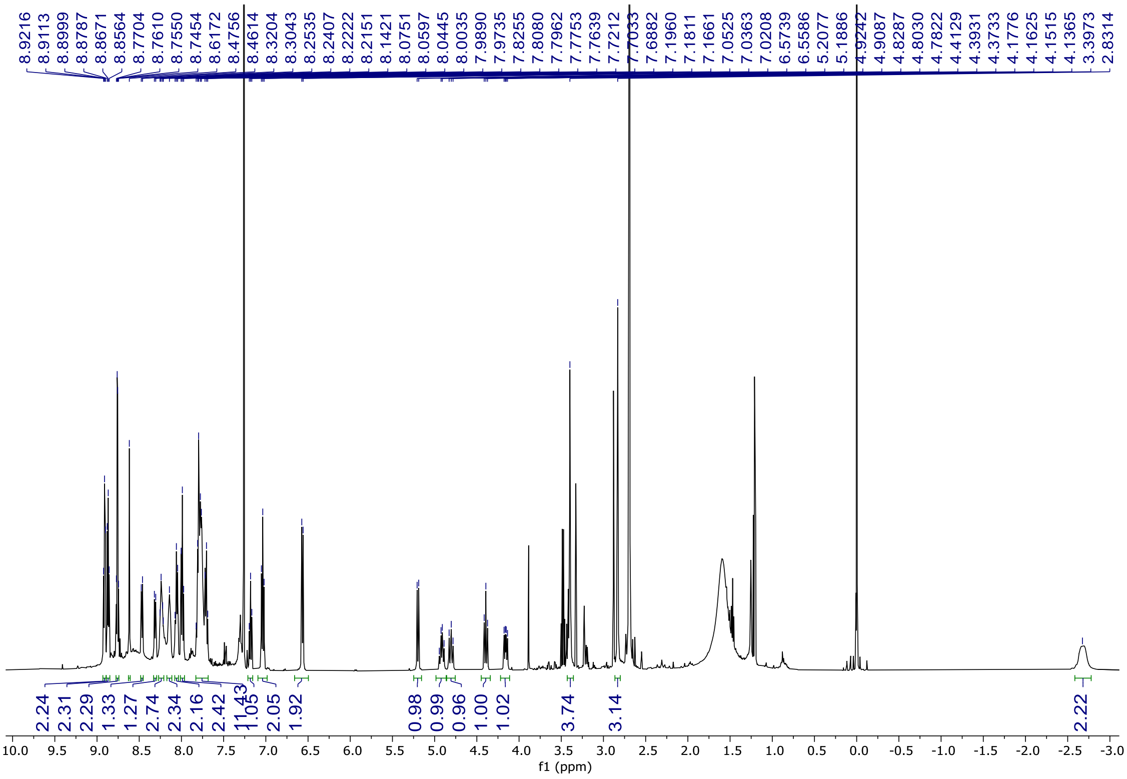

Figure S35. ^{1}H NMR spectrum of compound **7d** in $CDCl_3$.

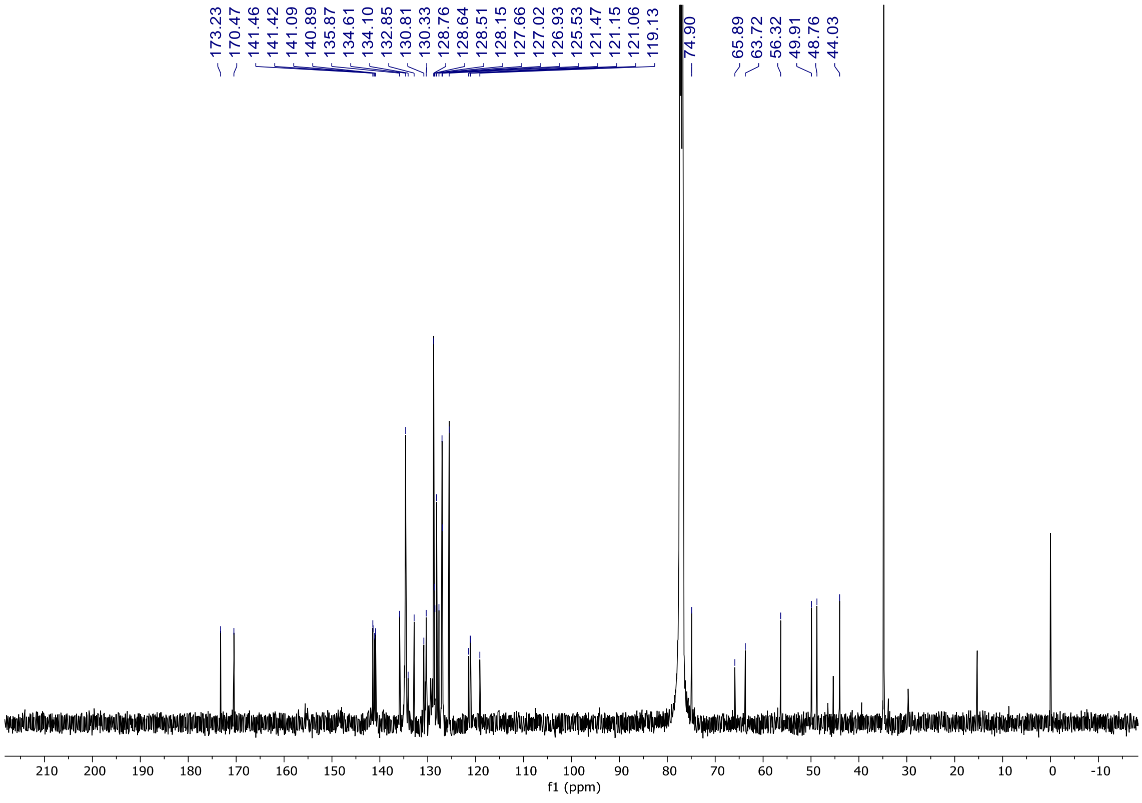

Figure S36. ^{13}C NMR spectrum of compound **7d** in $CDCl_3$.

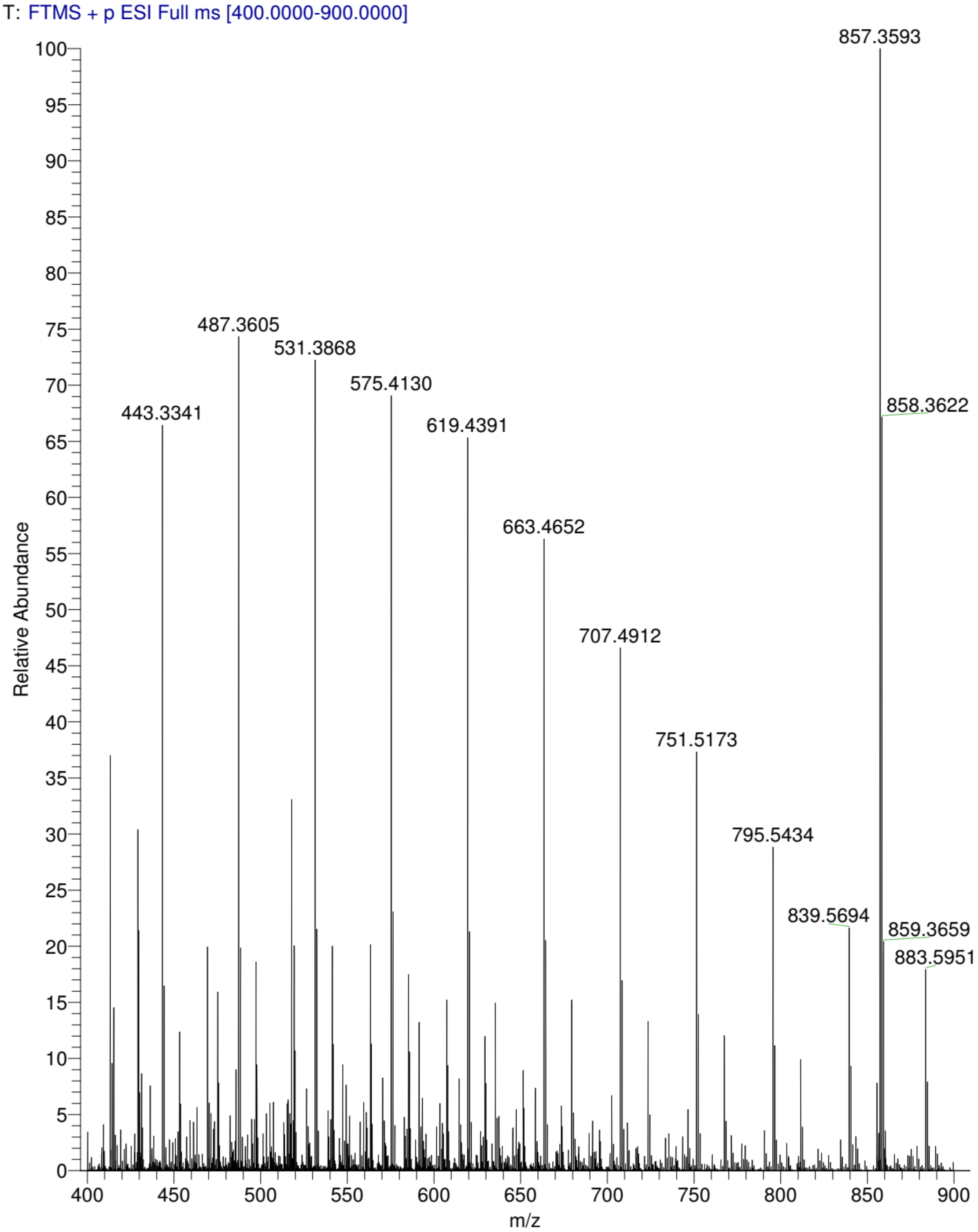

Figure S37. HRMS-ESI(+) spectrum of compound **7d**.

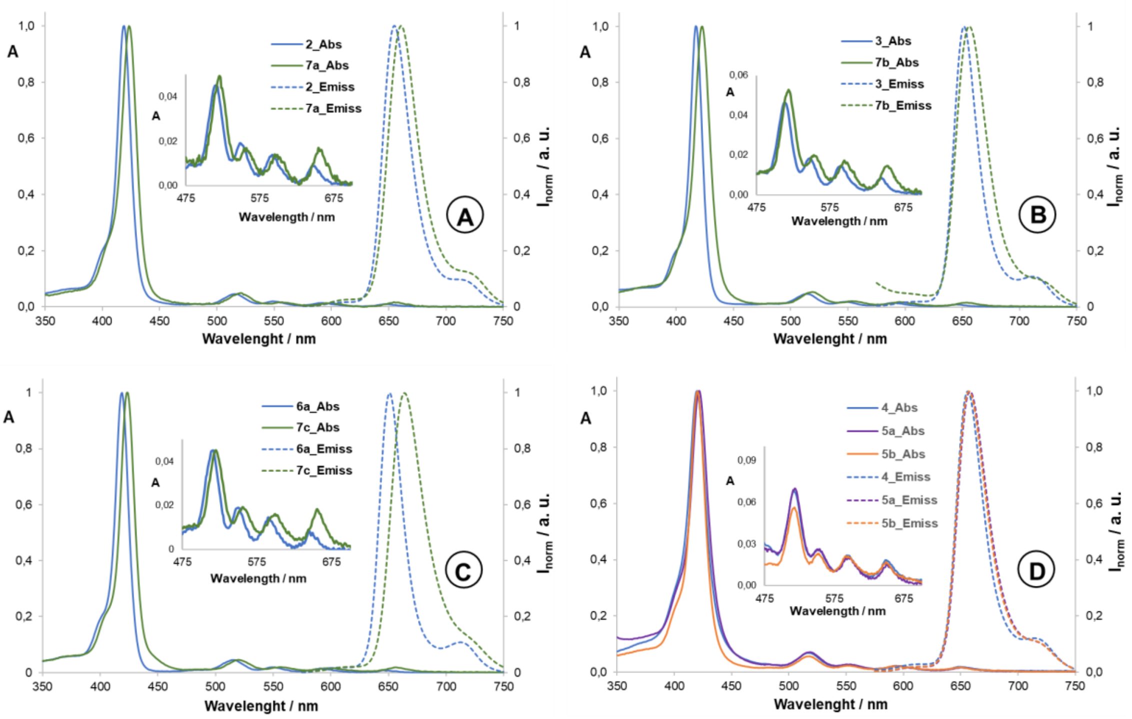

Figure S38. Absorption and normalized emission spectra of compounds **2** and **7a** (A), **3** and **7b** (B), **6a** and **7c** (C) and **4**, **5a** and **5b** in DMF at 298 K ([**2**] = [**3**] = [**4**] = [**5a**] = [**5b**] = [**6a**] = [**6b**] = [**7a**] = [**7b**] = [**7c**] = 2.5µM; $\lambda_{exc\mathbf{2}}$ = 419 nm; $\lambda_{exc\mathbf{7a}}$ = 423 nm; λ_{exc3} = 417 nm; $\lambda_{exc\mathbf{7b}}$ = 423 nm; $\lambda_{exc\mathbf{6a}}$ = 419 nm; $\lambda_{exc\mathbf{7c}}$ = 424 nm; $\lambda_{exc\mathbf{4}}$ = 420 nm; $\lambda_{exc\mathbf{5a}}$ = 422 nm; $\lambda_{exc\mathbf{5b}}$ = 420 nm). in DMF at 298 K. The inset shows the absorption at the Q bands region.
